# An organ boundary-enriched gene regulatory network uncovers regulatory hierarchies underlying axillary meristem initiation

**DOI:** 10.15252/msb.20145470

**Published:** 2014-10-30

**Authors:** Caihuan Tian, Xiaoni Zhang, Jun He, Haopeng Yu, Ying Wang, Bihai Shi, Yingying Han, Guoxun Wang, Xiaoming Feng, Cui Zhang, Jin Wang, Jiyan Qi, Rong Yu, Yuling Jiao

**Affiliations:** 1 State Key Laboratory of Plant Genomics, Institute of Genetics and Developmental Biology, Chinese Academy of Sciences, and National Center for Plant Gene ResearchBeijing, China; 2 College of Life Sciences, Capital Normal UniversityBeijing, China; 3 University of Chinese Academy of SciencesBeijing, China

**Keywords:** axillary meristem, gene regulatory network, organ boundary

## Abstract

Gene regulatory networks (GRNs) control development via cell type-specific gene expression and interactions between transcription factors (TFs) and regulatory promoter regions. Plant organ boundaries separate lateral organs from the apical meristem and harbor axillary meristems (AMs). AMs, as stem cell niches, make the shoot a ramifying system. Although AMs have important functions in plant development, our knowledge of organ boundary and AM formation remains rudimentary. Here, we generated a cellular-resolution genomewide gene expression map for low-abundance *Arabidopsis thaliana* organ boundary cells and constructed a genomewide protein–DNA interaction map focusing on genes affecting boundary and AM formation. The resulting GRN uncovers transcriptional signatures, predicts cellular functions, and identifies promoter hub regions that are bound by many TFs. Importantly, further experimental studies determined the regulatory effects of many TFs on their targets, identifying regulators and regulatory relationships in AM initiation. This systems biology approach thus enhances our understanding of a key developmental process.

## Introduction

Systems biology aims to explain development, physiology, and pathology based on modular networks of expression, interaction, regulation, and metabolism (Long *et al*, 2008; Wellmer & Riechmann, 2010). A major challenge in systems biology is to infer gene regulatory networks (GRNs). Gene expression is regulated in part by regulatory transcription factors (TFs) that bind to specific genomic regions. Emerging evidence from genome sequencing indicates that a significant portion of all eukaryote genomes encodes TFs; for example, ∼2,000 *Arabidopsis thaliana* genes encode TFs, more than many metazoan genomes (Riechmann *et al*, 2000). Each gene is likely regulated by multiple TFs, and each TF likely binds regulatory regions of multiple genes to activate or repress transcription. Furthermore, the majority of genes, including TF-encoding genes, show differential expression in various tissues and cell types in multicellular eukaryotes, including higher plants (Wang & Jiao, 2011). The combinatorial effect of tissue- and cell type-specific TF gene expression and the interaction between TFs and regulatory genomic regions of downstream genes results in qualitatively and quantitatively fine-tuned spatial and temporal gene expression. By integrating genomewide cellular-resolution expression and protein–DNA interaction (PDI) data, researchers can formulate hypotheses in biologically meaningful ways with higher confidence.

A first step in deciphering GRNs is the genomewide profiling of gene expression at cellular resolution. Several recently developed technologies, including laser microdissection, fluorescence-activated cell/nuclei sorting, and translating ribosome affinity purification, have extended transcriptome analysis in higher plants to the cellular resolution (Brady *et al*, 2007; Zhang *et al*, 2008; Jiao *et al*, 2009; Mustroph *et al*, 2009; Yadav *et al*, 2009, 2014; Deal & Henikoff, 2010; Jiao & Meyerowitz, 2010). Although the number of transcriptome profiles at cellular resolution remains far from comprehensive, an early glimpse of the cellular transcriptional landscape seems to be information-rich for properties of both the genes from which the transcripts are derived, and of the cell types.

Further deciphering of GRNs requires large-scale mapping of TFs and the regulatory genomic regions of their target genes. Recent advances in TF-centered genomewide assays of PDI, such as chromatin immunoprecipitation followed by sequencing (ChIP-seq), have broadly expanded our ability to delineate GRNs (Kaufmann *et al*, 2010; Ferrier *et al*, 2011). Although ChIP provides a very powerful method to identify PDIs *in vivo*, it is mostly limited to highly and/or broadly expressed TFs. In addition, ChIP-seq usually requires high-quality antibodies. These requirements make ChIP-seq less suitable for identifying PDIs specific to cell types that are difficult to enrich. By contrast, gene-centered yeast one-hybrid (Y1H) assays provide an alternative high-throughput approach for the systematic identification of PDIs (Vermeirssen *et al*, 2007a,b; Reece-Hoyes *et al*, 2011). Recent genomewide studies allowed large-scale detection of PDIs in *Arabidopsis* and created resources for genomewide Y1H assays (Mitsuda *et al*, 2010; Brady *et al*, 2011; Gaudinier *et al*, 2011; Ou *et al*, 2011).

The shoot apical meristem (SAM) contains a population of self-renewing stem cells located at the tip of the shoot apex. The SAM produces leaves and flowers from its peripheral zone and replenishes itself in the central zone. Cells between the meristem and the organ primordium undergo growth arrest, forming a discrete boundary domain that separates the forming organ from the SAM (Shuai *et al*, 2002; Aida & Tasaka, 2006; Rast & Simon, 2008).

Axillary meristems (AMs) form in the boundary region in seed plants (Hagemann, 1990; Schmitz & Theres, 2005; Domagalska & Leyser, 2011). AMs share the same developmental potential as the SAM, making the whole shoot a ramifying system. Our understanding of the fundamental developmental process of how the boundary establishes and how AMs initiate remains rudimentary. Because related mutants are often difficult to identify and these cells are very low in abundance, there is a clear demand for deciphering the underlying GRN as an alternative to genetic screens.

In this study, we combined cell type-specific genome expression analysis with genome-scale Y1H assays to initiate an experimental dissection of the GRN that acts in organ boundary cells. Our initial GRN allowed us to identify dominant signatures associated with boundary cells, system-level principles of gene regulation, and novel regulators and regulations controlling AM initiation and other boundary functions.

## Results

### Profiling boundary-specific gene expression using TRAP-seq

**Figure 1:**
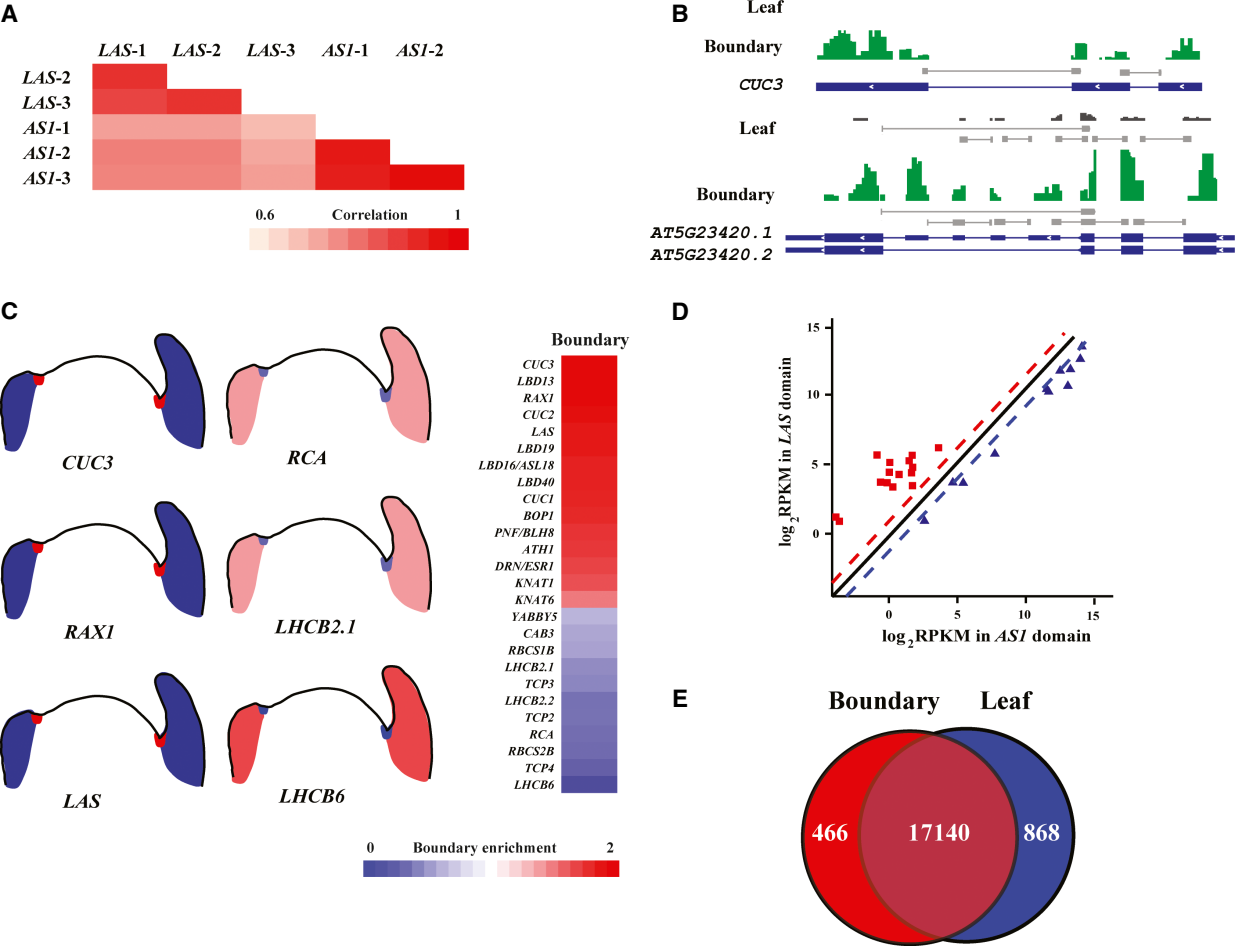
Quantification of boundary enrichment of gene expression by cell type-specific translatome analysis Pearson's correlation coefficients of translatome data from biological replicates for the *LAS* domain and the *AS1* domain.Translated mRNAs for boundary and leaf domains for a 1.9-kb region of chromosome 1 containing *CUC3* (*AT1G76420*) and a 1.7-kb region of chromosome 5 containing *HIGH-MOBILITY GROUP BOX 6* (*AT5G23420*). TAIR-annotated transcripts are shown as blue boxes at the bottom with ORFs highlighted as thick boxes. Selected reads covering exon–exon junctions are highlighted by short lines.Diagrams showing the boundary enrichment scores of previously characterized boundary-specific and leaf-specific genes. More examples are shown in the right with each row representing one gene. Genes were identified manually by searching PubMed abstracts followed by manual summarization of *in situ* and other types of data from each publication. Relative boundary enrichment scores were calculated by comparing boundary domain expression with leaf expression.Expression profiles of known boundary-enriched (red) and boundary-depleted (blue) genes.Venn diagram of cell domain-enriched genes that exhibited significant (≥ twofold with *P *<* *0.001) up-regulation. The number in the middle area indicates expressed genes without domain specificity.

To study cell type-specific gene expression in the leaf boundary region in the genome scale, we employed the TRAP-seq approach recently implemented by us and others (Mustroph *et al*, 2009; Jiao & Meyerowitz, 2010). In brief, we introduced a reporter line carrying the fusion of the large subunit ribosomal protein L18 with N-terminal His and FLAG epitope tags (*HF-RPL18*) under the control of the *pOp* promoter (Jiao & Meyerowitz, 2010) into driver lines expressing the chimeric TF LhG4 under the control of the *LATERAL SUPPRESSOR* (*LAS*) promoter, and under the control of the *ASYMMETRIC LEAVES1* (*AS1*) promoter. These driver lines were chosen because *pLAS::LhG4* has boundary region-specific activity (Goldshmidt *et al*, 2008), and *pAS1::LhG4* drives pOp reporter expression throughout emerging leaf primordia, but not in the SAM (Eshed *et al*, 2001) (Supplementary Fig S1). Cell type-specific expression of HF-RPL18 can efficiently incorporate epitope tags into polysomes for immunopurification of all translating cellular mRNAs. We immunopurified polysomes from seedlings at 7 days after germination (DAG), to isolate translating mRNA in the *LAS*-expressing organ boundary cells and *AS1*-expressing leaf primordia and cotyledon cells. Then, we used deep sequencing to map and quantify these mRNA samples. For each replicate, we obtained at least ∼20 million mapped 50-bp reads from each library and assayed three independent libraries for each cell type sample (Fig1A and Supplementary Table S1). Our previous study indicated that a sequence depth of > 10 million mapped reads is sufficient to reliably detect and measure rare, yet biologically relevant, mRNA species for the *Arabidopsis* genome (Jiao & Meyerowitz, 2010). The isolated cell type-specific transcripts from polysomes are likely translating and are collectively termed the translatome (Mustroph *et al*, 2009; Jiao & Meyerowitz, 2010).

Translatome sequencing resulted in a single-base resolution of transcript structures, as illustrated in Fig1B. The *CUP-SHAPED COTYLEDON3* (*CUC3*) TF gene is specifically expressed in the boundary domain (Vroemen *et al*, 2003; Hibara *et al*, 2006; Raman *et al*, 2008). Consistent with this, we identified 4,147 reads for *CUC3* in the boundary domain, in contrast to only 34 reads in the leaves. Translatome sequencing can also detect alternative splicing isoforms. Two annotated spliced isoforms of *AT5G23420* were both detected with low or modest expression levels in leaves or in the boundary domain, respectively, supported by reads that cross splice junctions (Fig1B).

As an additional step to ensure the quality and reliability of our data, we compared our translatome data set with published data, such as *in situ* hybridization results. We selected 26 genes with previously reported boundary-enriched expression or leaf-enriched expression and analyzed their enrichment levels based on our translatome data set. As shown in Fig1C and D, we detected the expected boundary enrichment or depletion for most genes and the comparisons validate the translatome profiling.

Cell type-specific translatomes showed qualitative and quantitative differences consistent with functional specialization. Using a transcript detection threshold of above 0.5 reads per kb of the transcript per million mapped reads of the transcriptome (RPKM), we identified 18,216 genes (66.44% of the genome) expressed in the boundary domain and 17,616 genes (64.25% of the genome) expressed in the developing leaves. We detected a small portion of the genome differentially expressed between the boundary domain and leaves (≥ twofold with adjusted *P *≤* *0.001), with 466 genes (1.70% of the genome) up-regulated and 868 genes (3.16% of the genome) down-regulated in the boundary domain (Fig1E). The domain-specific genes are listed in Supplementary Tables S2 and S3. The boundary-enriched genes included proteins with different functions, as listed in Supplementary Table S4.

We also compared our seedling boundary-enriched gene list with floral meristem boundary-enriched genes identified by a recent fluorescence-activated cell sorting study (Yadav *et al*, 2014). Among the 144 genes significantly enriched in the *LAS* domain, but not in the *CLVATA3* or the *KANADI1* domain in floral meristems (Yadav *et al*, 2014), we recovered 38 genes in our above-mentioned seedling *LAS*-domain-enriched genes (Supplementary Table S5), suggesting enrichment between these two gene lists. This enrichment is highly significant with a *P *<* *7.98E-36 using the hypergeometric test. Whereas several previously identified boundary-specific genes, such as *CUC3*, *LAS,* and *LIGHT-DEPENDENT SHORT HYPOCOTYLS4*, are among overlapping genes, our seedling data set includes adding boundary marker genes (Fig1C).

### Boundary cell properties uncovered through cell type-specific gene expression analysis

**Figure 2:**
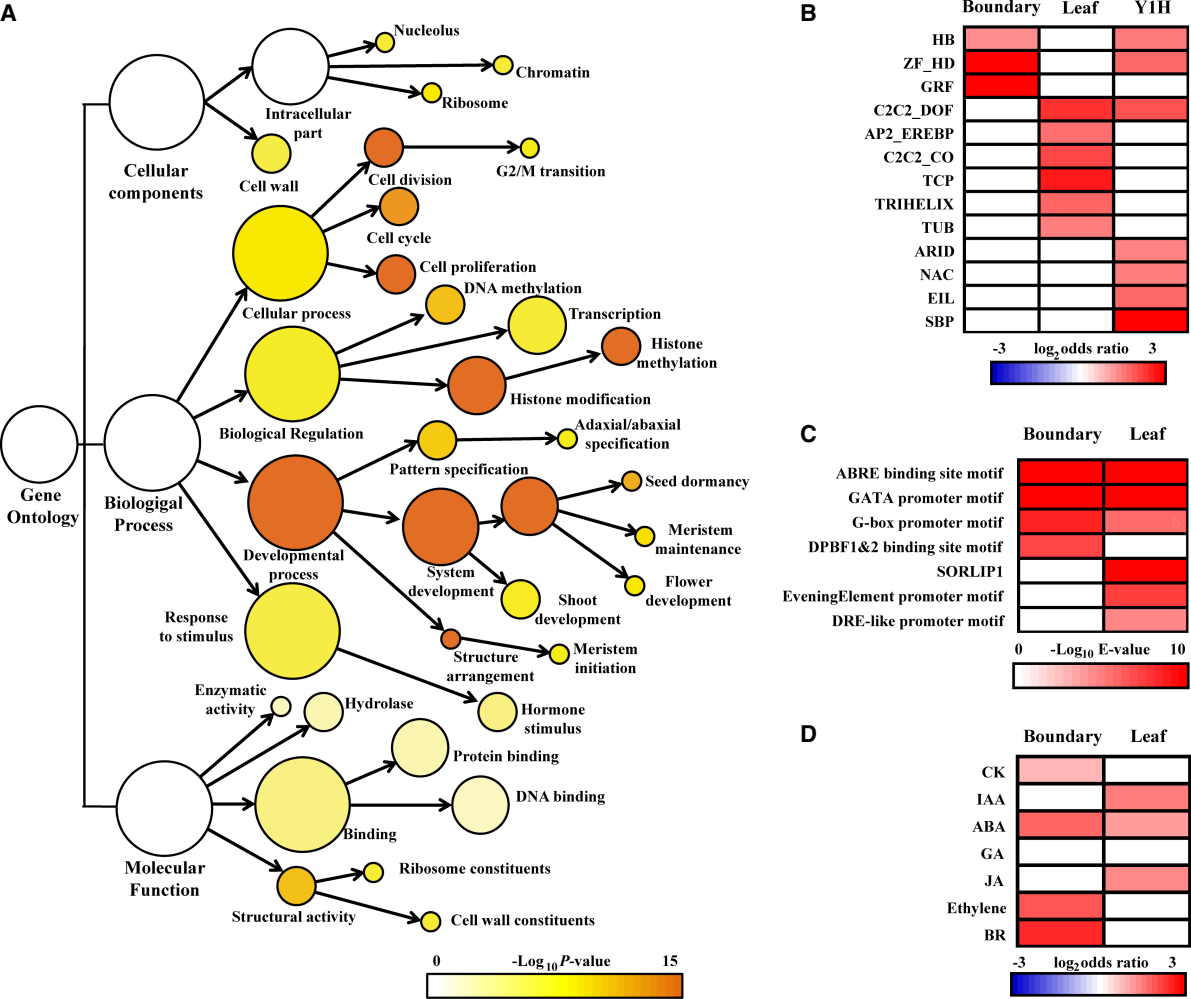
The spatially regulated translatome for boundary and axillary meristem (AM) formation Gene ontology (GO) analysis identified significantly over-represented (FDR adjusted *P *<* *0.01) gene categories for the boundary cell-specific transcripts. Color bar: significance levels for categories by hypergeometric test with FDR correction.Domain-specific and Y1H-enriched transcription factor (TF) families. Only significantly over-represented (*P *<* *0.05) families by hypergeometric test with FDR correction are colored.Domain-specific enriched known *cis*-elements in boundary and leaf domains. Only significantly over-represented (*E *< 10^−4^) classes are colored.Domain-specific enrichment (FDR adjusted *P *<* *0.05) of hormone-responsive genes in boundary and leaf domains. Red indicates enrichment and blue indicates depletion.

A comparison between the enriched and depleted translatomes for the boundary domain provided a wealth of genes with candidate developmental roles. Many gene ontology (GO) categories were enriched for the boundary domain, suggesting localized physiological functions (Fig2A and Supplementary Fig S2). First, we observed that the annotation of genes expressed preferentially in the boundary domain often corresponded to related physiological functions (Fig2A). For instance, we observed that ‘Meristem Initiation’ and ‘Organ Development’ were significantly enriched in organ boundary cells. In addition, many other GO terms, such as ‘DNA Binding’, ‘Hormone Stimulus’, ‘Histone Modification’, and ‘Cell Cycle’, were enriched, suggesting that these biological processes are associated with boundary domain cells. A detailed inspection indicated that it was mainly negative cell cycle regulators that were boundary-enriched. By contrast, the terms ‘Photosynthesis’, ‘Defense response’, and ‘Metabolism’ were depleted from boundary cells (Supplementary Fig S2), and also coincide with leaf functions. Genes localized to ‘Photosystem’ and ‘Chloroplast’ were also enriched in developing leaf cells, consistent with photosynthetic functions of leaves (Supplementary Fig S2).

Among other GO terms, we found that ‘Transcription’ was enriched in boundary domain cells. In addition, previous studies identified several TFs controlling boundary and AM formation. We therefore focused on TF-encoding genes (Supplementary Table S6) and identified TF families enriched in or depleted from organ boundary cells. We identified ZF-HD, GRF, and HB families enriched in organ boundary cells, and six other families, including the TCP family, depleted from organ boundary cells (Fig2B). Recent studies have shown that members of the TCP family are critical for leaf development (Koyama *et al*, 2010; Sarojam *et al*, 2010).

Through genes either co-expressed in or depleted from the boundary domain, we attempted to identify promoter DNA motifs associated with the boundary domain. We compared *cis*-element enrichment in the promoters of domain-specific genes and identified enrichment of a few *cis*-elements upstream of genes enriched in either category (Fig2C), suggesting that transcriptional activation and repression are equally important for boundary development. Among the *cis*-elements, ABRE-binding site, GATA box, and G-box were enriched in both categories, implying that their corresponding TF families occur in both boundary-enriched and boundary-depleted genes.

Hormones are key regulators of organogenesis. We also found that translating transcripts for hormone-responsive genes were enriched in organ boundary cells. We examined the sets of genes that respond to the phytohormones abscisic acid, auxin, brassinosteroid, cytokinin, ethylene, gibberellins, and jasmonic acid. The sources and lists of phytohormone-responsive genes are provided in Supplementary Table S7. Genes in these classes showed cell type-specific patterns of enrichment (Fig2D). In particular, we found genes responsive to brassinosteroid, ethylene, abscisic acid, and cytokinin were highly enriched in organ boundary cells, suggesting novel phytohormone activity centers. By contrast, genes responsive to auxin and jasmonic acid were enriched in leaf cells but depleted from boundary cells. This genomewide observation supports recently reported hormone signaling activities of the boundary domain. We, and others, identified the existence of an auxin minimum and a subsequent cytokinin pulse in the boundary domain, which are required for AM initiation (Wang *et al*, 2014a,b).

### Genomewide mapping of TF–DNA interactions by Y1H assays

**Figure 3:**
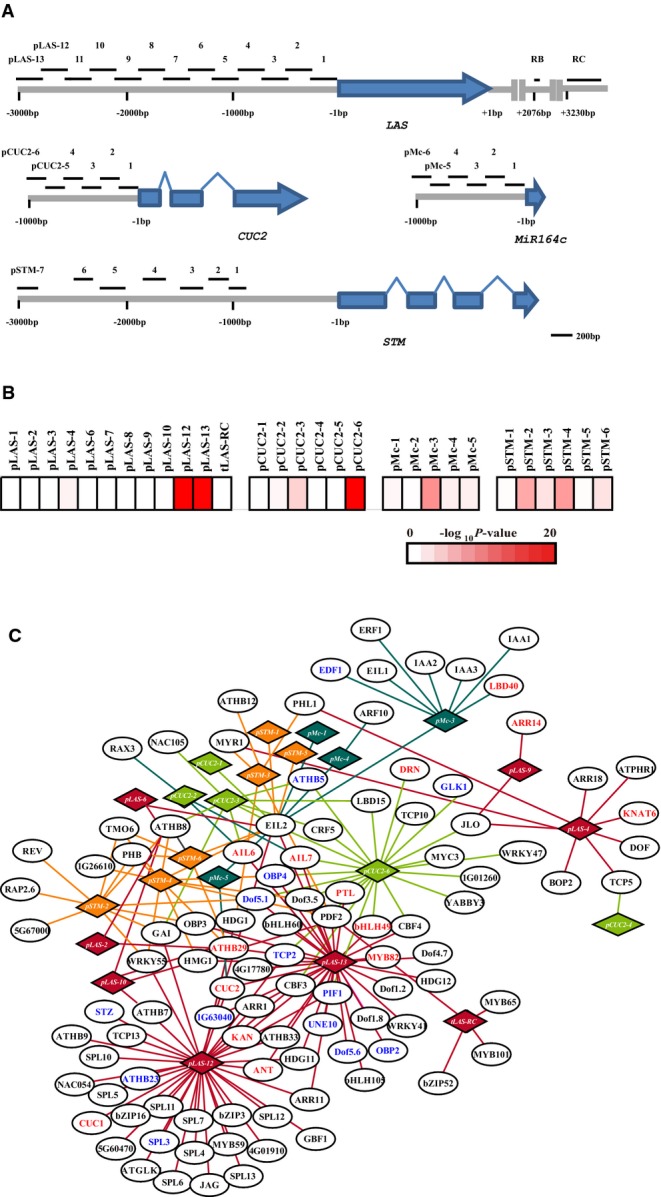
A boundary-enriched protein–DNA interaction (PDI) network Schematic of the genomic region subject to Y1H assay. TAIR-annotated ORFs are shown as blue boxes.PDI enrichment among tested genomic regions. Color bar: significance levels for genomic regions by hypergeometric test. Fragments with high background were excluded for this analysis.PDI network. Circle, transcription factor (TF), diamond; promoter fragment; edge, PDI. Boundary-enriched TFs are shown in red, and boundary-depleted TFs are shown in blue. Circles of the same color represent promoter fragments of the same gene. Source data are available online for this figure.

To dissect the GRN that regulates the boundary domain, we empirically mapped direct interactions between TFs and regulatory genomic regions by genomewide Y1H assays. We used a recently developed TF library (Ou *et al*, 2011), and added additional clones for boundary domain expressing TFs. This combined TF library containing 1,184 clones (listed in Supplementary Table S8) was subsequently used as protein prey in Y1H matrix assays. This library contains boundary-enriched TFs, as well as TFs with low expression in the boundary domain, to identify PDIs corresponding to both transcriptional activation and suppression. We next selected and cloned 34 regulatory genomic regions from promoters of TF genes that regulate boundary and AM formation, including *CUC2* (Hibara *et al*, 2006; Raman *et al*, 2008), *LAS* (Greb *et al*, 2003), and *SHOOT MERISTEMLESS* (*STM*) (Grbic & Bleecker, 2000; Long & Barton, 2000), and *MiR164c*, a miRNA targeting *CUC1* and *CUC2* with boundary-specific expression (Raman *et al*, 2008). Each fragment was 180–320 bp in length to ensure full transcriptional activation in yeast, because the majority of yeast promoters act within approximately 150–400 bp (Dobi & Winston, 2007). These fragments cover the 1,010-bp region upstream of *CUC2*, the 3,010-bp region upstream of *LAS*, the 3,000-bp region upstream of *STM*, the 1,010-bp region upstream of *MiR164c*, and two regions downstream of *LAS* (Fig3A and Supplementary Table S9).

We carried out pilot experiments by transforming TF plasmids DNA into haploid yeast bait strains, and mating each bait strain with TF-transformed yeast strains. Consistent with a previous study (Vermeirssen *et al*, 2007b), we found that the transformation strategy had both high coverage and high confidence, albeit at the cost of labor and expense. To ensure coverage and reliability of the resulting GRN, we chose the transformation strategy. We further tested a pooling strategy and found that limited pooling, with four TFs in each pool, gave results most similar to those obtained without pooling. We therefore performed all subsequent Y1H assays using the transformation strategy with limited pooling (Supplementary Fig S3).

From a total of 40,256 (fragment × TF) potential PDIs screened, we identified 180 PDIs between 103 TFs and 23 genomic regulatory regions (Fig3C and Supplementary Table S10). At least one interacting TF was identified for 67.7% of the regulatory genomic regions, and the majority of these regulatory genomic regions bound more than one TF (Fig3C). Also, 8.7% of TFs bound at least one regulatory genomic region. The majority (63.1%) of these identified TFs bound only once to a regulatory genomic region.

**Figure 4:**
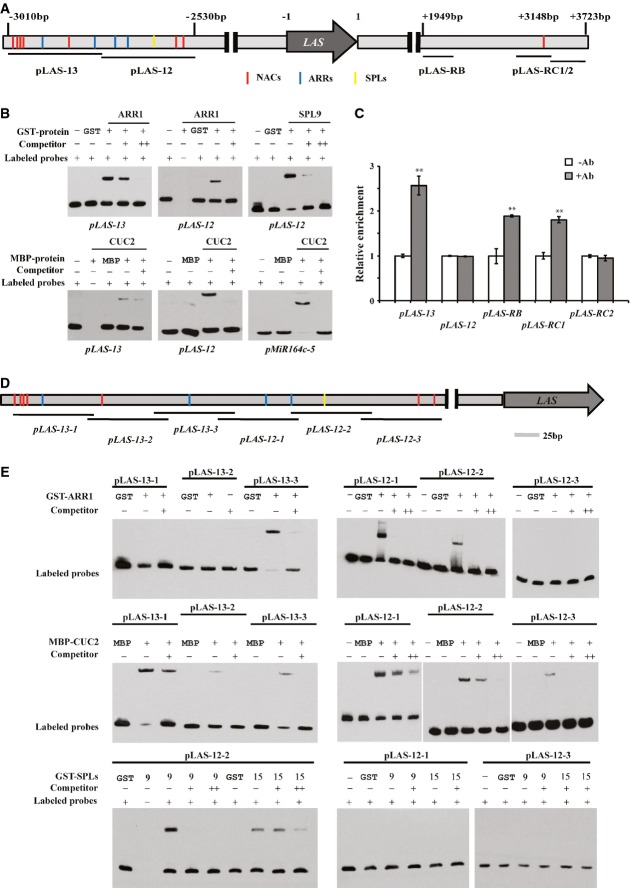
Validation of protein–DNA interactions (PDIs) Schematic of the *LAS* genomic region. Colored vertical lines indicate sites containing the consensus binding sequence: red, NAC binding box; blue, ARR binding box; yellow, SPL binding box. TAIR-annotated ORFs are shown as a thick gray box.Electrophoretic mobility shift assay (EMSA) validation of PDIs. The biotinylated DNA oligonucleotide probes are shown below each EMSA experiments. Recombined proteins that were used in EMSAs are indicated on top. Each lane represents no protein, protein tag, recombined protein, or recombined protein and unlabeled competitor DNA oligonucleotide probes, as individually labeled. Note, the weak interaction between maltose-binding protein (MBP)-CUC2 and fragment *pLAS-13* was further verified by dissecting *pLAS-13* into three fragments as shown in (E).*In planta* validation of PDIs using chromatin immunoprecipitation (ChIP) PCR. Five PCR fragments were designed for ChIP analysis. ChIP enrichment test by PCR shows binding of CUC2-GR-HA to the region near fragments pLAS-13, pLAS-RB, and pLAS-RC1. Error bars indicate s.d., and a double asterisk (**) represents *P*-value < 0.01.Schematic of the *LAS-12* and *LAS-13* genomic region in more detail. Binding boxes are indicated as above.Detailed dissection of transcription factor (TF) and DNA-binding regions using EMSA. Gels were labeled as in (B). Source data are available online for this figure.

Further confirmation of our identified PDIs came from independent electrophoretic mobility shift assays (EMSAs). B-type ARABIDOPSIS RESPONSE REGULATOR1 (ARR1), CUC2, and SQUAMOSA PROMOTER-BINDING PROTEIN-LIKE (SPL) family members were retrieved in the Y1H screen. Using recombinant glutathione S-transferase (GST)-ARR1, maltose-binding protein (MBP)-CUC2, GST-SPL9 and GST-SPL15, and regulatory genomic region fragments of *LAS* and *MiR164c* identified by Y1H (Figs3A and 4A), we found that the recombinant TF proteins were able to bind to the DNA fragment and cause mobility shifts (Fig4B). Addition of unlabeled DNA of identical sequence competed with the binding; also, the mobility shift was not observed when DNA fragments were incubated with GST or MBP alone, indicating that these PDIs were specific (Fig4B). Both CUC2 and ARR1, which activate *LAS* expression, and SPL9 and SPL15, which suppress *LAS* expression, interact with the overlapping pLAS-12 and pLAS-13 genomic fragments in Y1H assays. However, more careful dissection of this region using ∼90-bp tiling fragments identified a 480-bp region bound by CUC2 and a 230-bp region bound by ARR1 with a 230-bp overlap (Fig4D and E). By contrast, both SPL9 and SPL15 interact with a 50-bp region that contains an SPL-binding motif and is also bound by ARR1 and CUC2 (Fig4D and E),

To further determine whether the PDIs that we identified occur *in planta*, we used ChIP coupled with PCR to examine the interactions of CUC2 with the *LAS* gene. Using ChIP-PCR, we verified the CUC2 interaction with the pLAS-13 region (Fig4C), although the overlapping pLAS-12 region with weaker Y1H assay score was not enriched by ChIP. A recent study demonstrated the importance of two 3′ genomic regions, termed regions B and C, which are sufficient to guide boundary-specific expression (Raatz *et al*, 2011). Although we were not able to include region B in our Y1H assay due to its high auto-activation activity, we found direct binding of CUC2 with the region B in ChIP assays (Fig4C). We also detected interaction of CUC2 with the region C (Fig4C), which was not recovered using Y1H assays.

### Properties of TFs involved in PDIs

**Figure 5:**
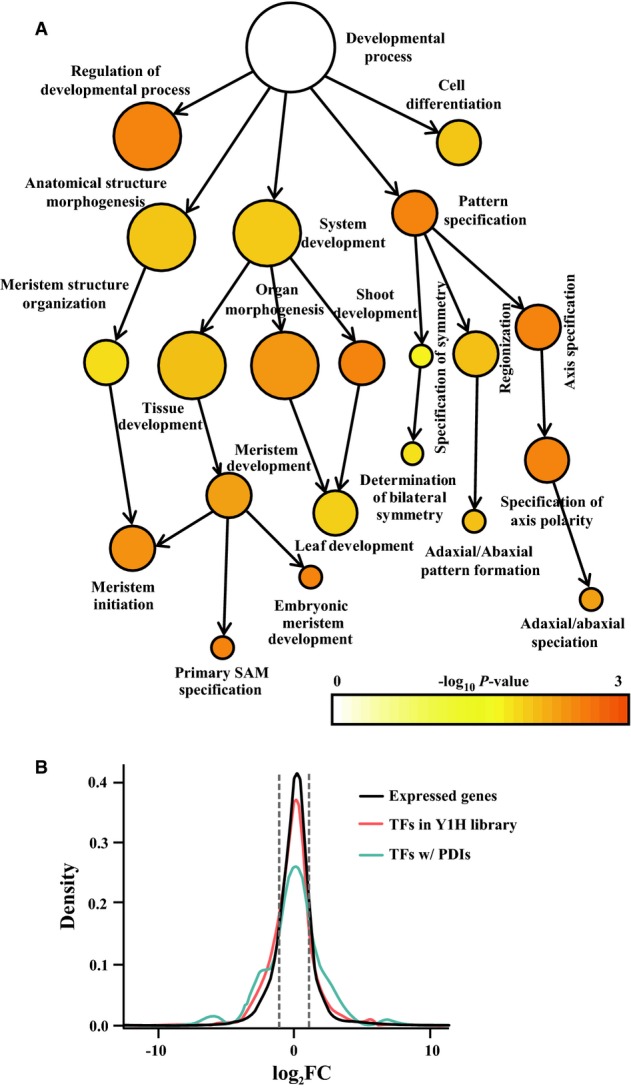
Properties of the boundary-enriched protein–DNA interaction (PDI) network Gene ontology (GO) analysis identified significantly over-represented (FDR adjusted *P *<* *0.05) gene categories for the transcription factors (TFs) involved in the PDI network. Color bar: significance levels for categories by hypergeometric test with FDR correction.A boundary enrichment/depletion level distribution of all expressed genes, TF-encoding genes covered by our Y1H library, and TF-encoding genes associated with PDIs. Gray vertical lines show boundary/leaf ratios of 0.5 and 2, which were used as cutoffs for boundary domain depletion and enrichment, respectively.

For the TFs associated with one or more PDIs identified in this work, many GO categories were enriched (Fig5). When compared to the TFs included in our Y1H library, we observed that the annotation of TFs with identified PDIs corresponds, in many cases, to related physiological functions. For instance, we observed ‘Meristem Initiation’, ‘Primary SAM Specification’, ‘Leaf Development’, and ‘Polarity Specification of Adaxial/Abaxial Axis’ were significantly enriched in the TFs involved in PDIs. Notably, the enriched GO categories in these TFs associated with PDIs were quite similar to the GO categories enriched in the boundary domain (Fig2A).

We also analyzed enrichment of members of each TF family in PDI-associated TFs identified in this study. We found that HB and ZF-HD families of TFs, which were enriched in the boundary domain based on expression, were also enriched in PDI-associated TFs (Fig2B). In addition, the boundary domain depleted C2C2-DOF family was enriched in PDI-associated TFs. Additionally, the SBP, ARID, EIL, and NAC families were also enriched in PDI-associated TFs.

In fact, we found that the PDI-associated TFs are enriched in transcripts enriched or depleted from the boundary domain. Only 8.7% of the TFs are associated with at least one PDI, but 13.6% of boundary region-enriched TFs and 13.6% of boundary region-depleted TFs bound to the regulatory genomic regions we tested. To better illustrate the enrichment of PDI-associated TFs in organ boundary-enriched and boundary-depleted genes, we carried out Kernel density estimate analysis with translatome data as the background and found that PDI-associated TFs have obvious differential expression patterns, as seen from shoulders on both sides of the density curve (Fig5B).

### Genomic regions that serve as regulatory hubs

Biological networks are characterized by a scale-free connectivity distribution containing hubs with many connections and a large number of nodes with one or a few connections (Barabasi & Oltvai, 2004; Albert, 2007). In the organ boundary domain GRN, we observed that one genomic region (covering fragments pLAS-12 and pLAS-13) upstream of *LAS* and one genomic region (pCUC2-6) upstream of *CUC2* connected to a large number of TFs (Fig3B and C). These regulatory genomic regions may serve as hubs and be subject to more complex regulation (Nelson *et al*, 2004). Notably, these putative regulatory hubs are bound by TFs positively regulating expression and TFs negatively regulating expression (Fig3C). In addition, these regulatory genomic hubs can be distant from the start codon (Fig3A). Finally, these hubs control important downstream organ boundary regulators, which are likely also hubs within the GRN (Aida *et al*, 1997; Greb *et al*, 2003).

### Inferring GRN by data integration

**Figure 6:**
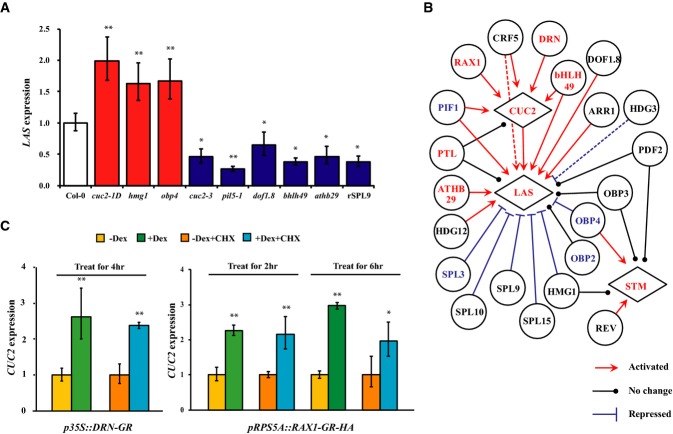
Regulatory relationships of a protein–DNA interaction (PDI) sub-network Real-time RT–PCR analysis of target gene expression in wild-type and in transcription factor (TF) mutant or over-expression lines. Error bars indicate s.d., a double asterisk (**) represents *P*-value < 0.01, and an asterisk (*) represents *P*-value < 0.05 between wild-type and a mutant or over-expression line.PDIs that result in activating (red line), repressive (blue line), and no effect (black line) in target expression were determined using qPCR of the TF and its target as shown in (A) and in Supplementary Fig S5. Dotted lines represent referred interaction from homologous TFs. Boundary-enriched TFs are shown in red, and boundary-depleted TFs are shown in blue.Real-time qRT–PCR analysis of *CUC2* using the *p35S::DRN-GR* inflorescences and analysis of *CUC2* in *pRPS5A::RAX1-GR-HA* seedlings before and after Dex treatment or simultaneous Dex and cycloheximide treatment. Vertical axis indicates relative mRNA amount compared with the amount before Dex treatment, or in cycloheximide treatment only. Error bars indicate s.d., a double asterisk (**) represents *P*-value < 0.01, and an asterisk (*) represents *P*-value < 0.05.

To assess the regulatory potential of our inferred GRN, we used an independent modeling approach to predict the regulatory potential of randomly selected PDIs. To this end, we employed qRT–PCR to analyze the effects of mutations and over-expression of TFs on the expression of their putative target genes. As shown in Fig6A, examination of the over-expressing allele *cuc2-1D*, which contains a single point mutation in the miRNA target site (Larue *et al*, 2009), and the loss-of-function allele *cuc2-3* indicated that CUC2 activates the expression of *LAS*, which is consistent with our predicted regulatory network based on our translatome data and published *in situ* hybridization results (Hibara *et al*, 2006; Raman *et al*, 2008). Analysis of a T-DNA insertion mutant of a novel HIGH-MOBILITY GROUP (HMG) family TF-encoding gene (*At1 g76110*, *HMG1*) indicated that HMG1 negatively regulates *LAS* expression (Fig6A). In total, we examined 30 putative regulatory interactions in 19 TF mutant alleles and seven TF over-expression alleles using inflorescence tissue, which is enriched in boundary domain cells. Among these 30 regulatory interactions, 15 (50.0%) involved activation, 7 (23.3%) involved repression, and the remaining 8 (26.8%) did not show clear *in planta* regulation of putative target expression (Fig6B and Supplementary Fig S4). After plotting expression values of a TF and its target gene in the wild-type and in a TF mutant/over-expression allele, we estimated the degree of activation or repression by fitting a line using weighted least squares regression across replicates (Supplementary Figs S4 and S5), where the slope of the line predicts the degree of activation or repression and the *P*-value represents the confidence level for the regulation (Brady *et al*, 2011). As shown in Supplementary Fig S5, CRF5 and CUC2 strongly activate their targets (*CUC2* and *LAS*, respectively).

We further used a chemically inducible line to independently test the inferred regulatory interactions. DORNROSCHEN (DRN, also known as ENHANCER OF SHOOT REGENERATION1) bound a *CUC2* promoter region in the Y1H assay; therefore, we explored the ability of DRN to elicit *CUC2* expression *in vivo*. To this end, we generated a line in which a DRN–glucocorticoid receptor (GR) fusion protein is expressed from the constitutive 35S promoter. Nuclear translocation of the DRN-GR fusion protein can be specifically triggered by treatment with the steroid hormone dexamethasone (Dex). DRN activation in *p35S::DRN-GR* plants mimics the *DRN* over-expression phenotype (Banno *et al*, 2001; Kirch *et al*, 2003). We measured the effect of DRN activation in *p35S::DRN-GR* plants on the expression of *CUC2* by qRT–PCR. DRN activation resulted in rapid elevation of *CUC2* mRNA levels, within 4 h of DRN induction, even in the presence of the protein synthesis inhibitor cycloheximide (Fig6C). Our results not only support induction of *CUC2* expression by DRN, but also strongly suggest that induction of *CUC2* does not require *de novo* protein synthesis and that *CUC2* is likely a direct target of DRN, which is consistent with the Y1H assay. Using the same strategy, we also generated an inducible *REGULATOR OF AXILLARY MERISTEMS1* (*RAX1*) over-expression line under the ubiquitous *RIBOSOME PROTEIN 5A* (*RPS5A*) promoter. After Dex induction in *pRPS5A::RAX1-GR-HA* plants, we found that *CUC2* gene expression increased within 2 h, and this induction was unaffected when cycloheximide was added. The results show that RAX1 can directly activate *CUC2* expression *in vivo*, which supports and extends the PDI identified by Y1H.

We next asked whether boundary-enriched TFs tend to activate target genes in the same domain. All the regulatory genomic regions tested in this study by the weighted least squares regression approach correspond to genes with enriched expression in the boundary domain (*CUC2*, *LAS,* and *STM*). We considered a regulation as regenerative if a TF is enriched in the boundary domain and it is within a transcriptional activation PDI. A regulation was also considered regenerative if a TF is depleted from the boundary domain and it is within a transcriptional suppression PDI. We consider a regulation as degenerative if a TF is enriched in the boundary domain but it is within a transcriptional suppression PDI, or a TF is depleted from the boundary domain but it is within a transcriptional activation PDI. Using such criteria, we found eight regenerative and three degenerative regulatory interactions (Fig6B). These regenerative regulation interactions include both transcriptional activation (6) and transcriptional suppression (2). There were six additional PDIs resulting in target activation and five PDIs resulting in target suppression without significant TF gene enrichment in the boundary domain.

### Regulators of GRN hubs control AM initiation and other boundary domain functions

We reasoned that the regulatory genomic region hubs integrate regulation from multiple upstream TFs, and therefore, manipulation of upstream TF expression should partially mimic mutation in, or over-expression of, the downstream hub gene, depending on regulatory interaction. To test this, we analyzed morphological phenotypes using mutants and over-expression lines and searched the literature. In total, 25 mutants and transgenic plants, corresponding to 22 TF genes that bound regulatory genomic region hubs, were analyzed for AM and leaf morphological phenotypes (Supplementary Table S11). Boundary domain phenotypes, including AM initiation, boundary fusion, cotyledon number variation, and leaf serration, were associated with 7 (31.8%) TF genes.

**Figure 7:**
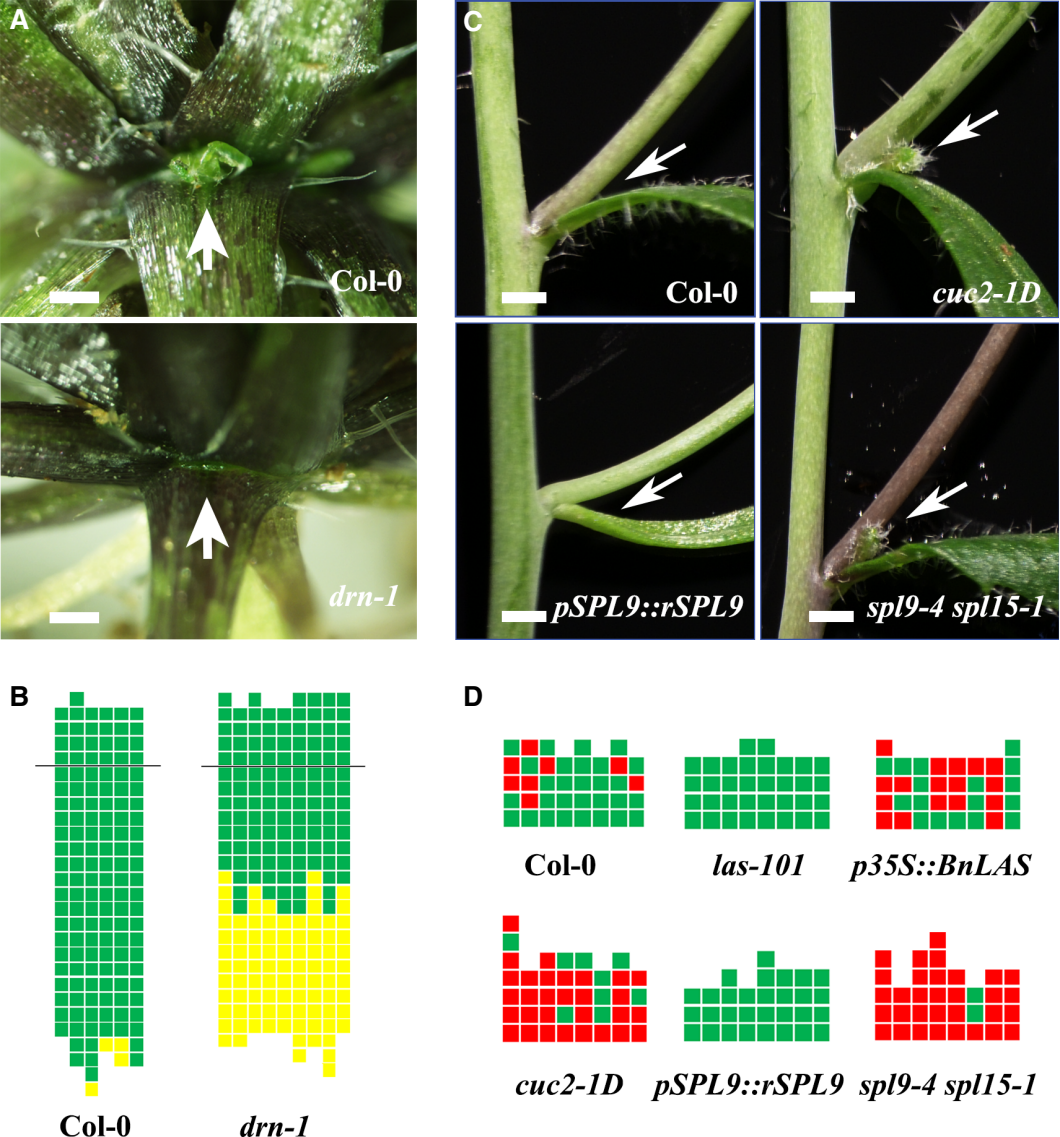
Phenotypic characterization of new mutants affecting axillary meristem (AM) initiation Close-up of rosette leaf axils in Col-0 wild-type and *drn-1* showing the presence (arrow) and absence (arrow) of an axillary bud, respectively. Scale bars, 5 mm.Schematic representation of axillary bud formation in leaf axils of Col-0 wild-type plants and the *drn-1* mutant plants. The thick black horizontal line represents the border between the youngest rosette leaf and the oldest cauline leaf. Each column represents a single plant, and each square within a column represents an individual leaf axil. The bottom row represents the oldest rosette leaf axils, with progressively younger leaves above. Green indicates the presence of an axillary bud, and yellow indicates the absence of an axillary bud in any particular leaf axil.Comparisons of cauline leaf axils of Col-0 wild-type, *cuc2-1D*, *pSPL9::rSPL9*, and *spl9-4 spl15-1*. Arrows point to accessory buds. Scale bars, 2.5 mm.Schematic representation of accessory bud formation in leaf axils of Col-0 wild-type, the *las-101* mutant, a *p35S::BnLAS* over-expression line, the *cuc2-1D* over-expression mutant, a *pSPL9::rSPL9* over-expression line, and the *spl9-4 spl15-1* mutant. Only cauline leaf axils are shown. Green indicates the presence of an axillary branch but lack of an accessory bud, and red indicates the presence of an accessory bud.

Based on our inferred regulatory network, DRN activates *CUC2* expression through direct PDI. We found clear AM initiation defects in the *drn-1* mutants (Fig7A and B), in which AMs could no longer initiate in the first ∼10 rosette leaves, a phenotype similar to the loss-of-function *cuc2-3* mutants (Hibara *et al*, 2006; Raman *et al*, 2008). In addition, it was previously reported that the cup-shaped cotyledon phenotype and other cotyledon number variations were observed at low penetrance in *drn* and *cuc2* mutants (Aida *et al*, 1997; Chandler *et al*, 2007). Taken together, these results indicate that DRN regulation of *CUC2* expression is likely biologically meaningful for AM initiation and cotyledon formation.

In addition, we identified *PTL* as a putative negative regulator of *CUC2* expression. Because *PTL* is enriched in the boundary domain in addition to its expression in leaves, *ptl* mutations should cause qualitative and quantitative expansion of *CUC2* expression. Indeed, we observed a serrated leaf margin phenotype in the *ptl-1* mutants (Supplementary Fig S6A), a phenotype very similar to that of *cuc2-1D*, in which the *CUC2* expression domain is enlarged (Larue *et al*, 2009). The antagonistic actions of *PTL* and *CUC2* support a recent genetic analysis (Lampugnani *et al*, 2012; Nahar *et al*, 2012). However, we did not find a clear change in *CUC2* expression in the inflorescence of *ptl* mutants (Fig6C), which may reflect the limitations of using the inflorescence to represent boundary domain cells. Similar to *ptl-1*, an *hmg1* mutant line also showed a leaf margin phenotype (Supplementary Fig S6A). HMG1 directly suppresses *LAS* expression (Fig6B), so this phenotype supports the view that *LAS* regulates leaf margin development (Busch *et al*, 2011).

The boundary domain GRN identifies CUC2 and SPL as positive and negative regulators of *LAS* expression. *LAS* functions as a central regulator of AM initiation (Greb *et al*, 2003). Consistent with the identification of CUC2 as a positive regulator of *LAS*, previous studies reported reduced *LAS* expression and AM initiation defects in *cuc2* mutants (Hibara *et al*, 2006; Raman *et al*, 2008). In addition to enhanced *LAS* expression in the *CUC2* over-expressing *cuc2-1D* mutants (Fig6A), we observed enhanced production of accessory meristems, which are additional AMs occasionally formed in wild-type plants (Fig7D), in cauline leaf axils in *cuc2-1D* (Fig7C and D). *Arabidopsis* plants weakly over-expressing *Brassica napus LAS* (*BnLAS*) (Yang *et al*, 2011), also showed similar over-production of accessory meristems (Fig7D), confirming that this phenotype is associated with ectopic activation of *LAS*.

*SPL* genes represent a plant-specific TF family. Recent studies have shown that *SPL* genes in rice and maize are responsible for panicle complexity and the establishment of boundaries (Chuck *et al*, 2010; Jiao *et al*, 2010; Miura *et al*, 2010). The orthologous genes of these two SPLs in *Arabidopsis* are *SPL9* and *SPL15* (Xie *et al*, 2006). Studies on these genes indicated that SPL activity inhibits initiation of new leaves at the SAM and affects organ size (Wang *et al*, 2008). To test whether SPL suppression of *LAS* expression has biological relevance to AM initiation, we analyzed AM initiation in the *spl9-4 spl15-1* mutant and in a *pSPL9::rSPL9* line containing mutations in the target sites for miR156 and miR157 (Wu & Poethig, 2006; Wang *et al*, 2008; Li *et al*, 2012). We observed more accessory meristems in cauline leaf axils in *spl9-4 spl15-1* mutants, similar to *p35S::BnLAS* and *cuc2-1D* (Fig7C and D). In contrast, plants containing a *pSPL9::rSPL9* transgene, as well as *las-101* mutants, lack accessory shoots (Fig7D). Taken together, these data support the idea that *SPL* suppression of *LAS* expression controls AM initiation in cauline leaf axils.

Another PDI we identified pointed to HDG12 as a positive regulator of *LAS* expression. In *hdg12* mutants, we found reduced *LAS* expression, as well as defective cotyledon development with incomplete penetrance, including tricots and partially fused cotyledons (Supplementary Fig S6B and D). Inappropriate cotyledon development in *hdg12* mutants also resulted in alterations of leaf phyllotaxy and sometimes leaf fusion (Supplementary Fig S6C). Such phenotypes have been previously found in several boundary defective mutants (Aida *et al*, 1997; Chandler *et al*, 2007), implying that *HDG12* may affect cotyledon development by regulating the boundary GRN, including *LAS*.

## Discussion

### Systems developmental biology for understanding organ boundary and AM formation

Unlike most animals, plants can initiate new organs during post-embryonic development. Organ boundaries separate lateral organs from the stem cell-containing meristems. In addition, AMs, as branch meristems, initiate from leaf boundaries to give rise to a new cycle of growth and development and thus make the shoot a ramifying system. This key characteristic of plant development leads to a major distinction between animal and plant development and is a central mechanism that allows plants to adapt to their changing local environments. Unfortunately, our understanding in this field of great importance remains rudimentary, largely due to difficulties in genetic screening for mutants deficient in boundary or AM formation in model plants, such as *Arabidopsis* (Rast & Simon, 2008). Nevertheless, forward and reverse genetic studies over the past two decades have identified several key TFs affecting boundary specification and AM initiation, implying that a complex GRN underlies boundary specification and AM initiation.

**Figure 8:**
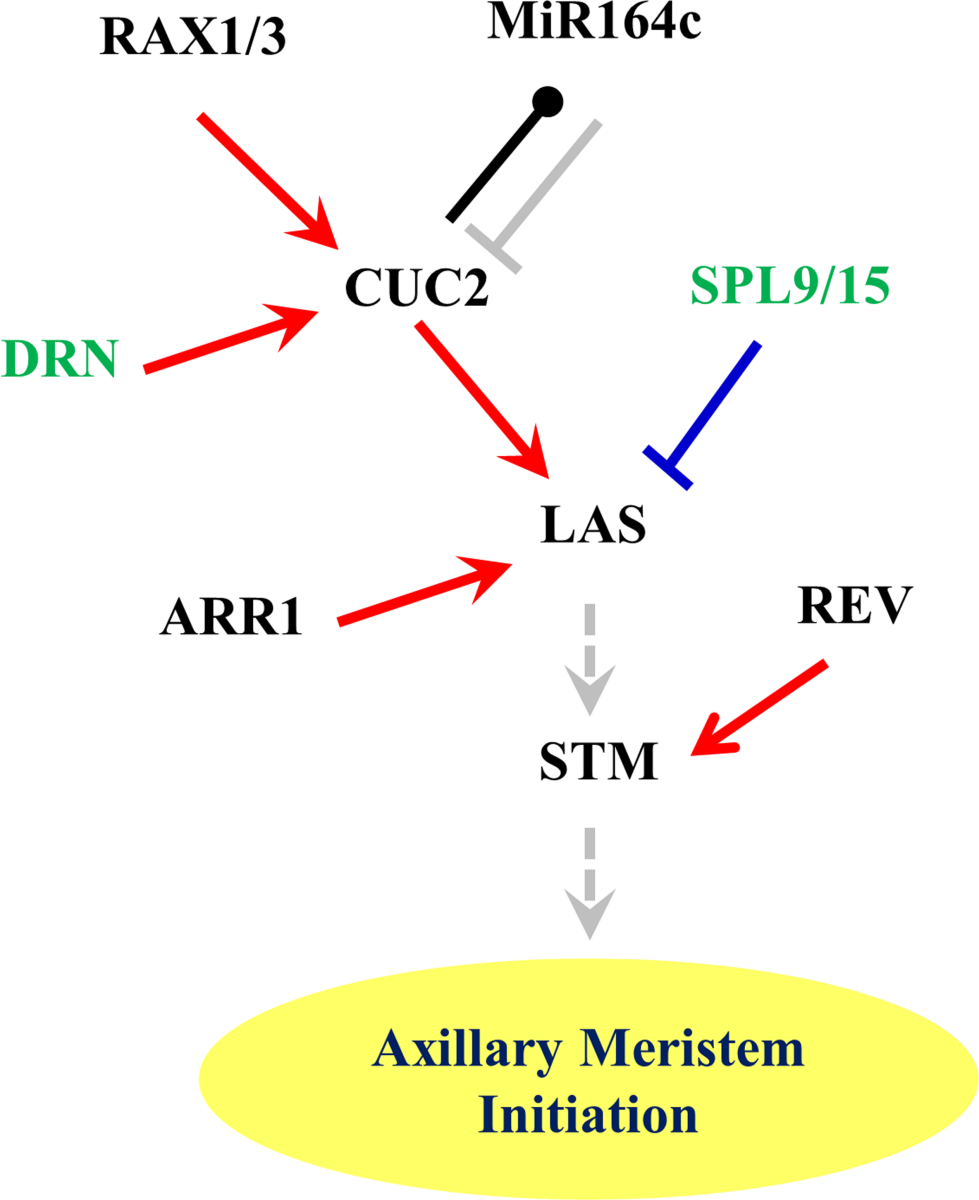
Summary of known and newly identified regulators and regulatory relationships controlling AM initiation Gray solid line, known direct interaction between miRNA and targeting mRNA; gray dotted line, known genetic interaction; red arrow, activating PDI identified in this study, blue bar, repressive PDI identified in this study; black line, PDI identified in this study, unknown regulatory relationship. New regulators of AM initiation are shown in green.

Using a systems biology approach, we integrated cell type-specific gene expression and PDIs on a genomewide scale to examine organ boundary and AM formation. Because boundary cells have very low abundance, we chose a Y1H-based assay instead of a ChIP-based assay to reliably detect PDIs. Complementary to reductionist studies, systems biology offers the potential to provide a comprehensive understanding of the causal relationships underlying boundary and AM formation. To this end, developmental biology networks derived from system-wide studies promise to link isolated genes and regulatory mechanisms identified by reductionist studies into a framework containing causal relationships and to allow formulation of new predictions (Long *et al*, 2008; Lander, 2011). Indeed, our derived GRN links most previously isolated key regulators into a network of direct interactions and regulation (Fig8). For example, the direct activation of *CUC2* by RAX1 and RAX3 and the direct activation of *LAS* by CUC2 extend and support previous genetic analysis (Hibara *et al*, 2006; Raman *et al*, 2008). The direct binding of CUC2 to the *MiR164c* promoter identifies an additional reciprocal regulation between *CUC* genes and *MiR164* miRNAs (Laufs *et al*, 2004; Mallory *et al*, 2004). The direct activation of *LAS* by ARR1 provides a molecular link between AM initiation and cytokinin signaling and extends our recently reported requirement for cytokinin in AM initiation (Han *et al*, 2014; Wang *et al*, 2014b).

In addition, we identified new players regulating AM initiation and boundary formation. Our detailed analysis of mutants and over-expression lines confirmed that AM initiation was compromised in *drn-1* mutants (Fig7A and B), likely due to DRN activation of *CUC2* (Fig6D). We also showed that AM initiation was ectopically activated in the *spl9-4 spl15-1* line and that boundary formation was affected in a *SPL9* over-expressing line (Fig7C and D). Taking our results together, our work shows that employing a top-down systems approach greatly speeds our understanding of AM initiation and boundary specification by identifying meaningful new components and new network interactions.

### New views of boundary and AM development

By combining cell type-specific transcription and genomewide PDIs, we were able to recapitulate and extend previously identified work on AM and boundary formation. First, we confirmed boundary enrichment or depletion of a large number of genes (Fig1C). Furthermore, independent GO analysis of genes enriched in the boundary domain and GO analysis of TFs bound to promoters of key regulators of boundary specification and AM initiation separately identified meristem-related GO functions (Figs2A and 5A). Additionally, we provided genome-scale support for the recent finding that a low auxin niche is required for AM initiation (Wang *et al*, 2014a,b), which is followed by a cytokinin signaling pulse (Han *et al*, 2014; Wang *et al*, 2014b).

More importantly, our systems analysis identified numerous examples from boundary and AM formation in which a GRN makes possible new views of the properties of cell types and their development not evident from previous, reductionist approaches. Our results showed that cell cycle regulation, transcriptional regulation, epigenetic regulation, and cell wall homeostasis all likely affect boundary and AM formation (Figs2A and 5A). In addition to auxin and cytokinin, we found another five major phytohormones positively or negatively associated with this developmental process (Fig2D), indicating new directions in the study of boundary and AM formation. Our studies also identified a few TF families enriched in the boundary domain and/or enriched with TFs participating in PDIs (Fig2B). TFs are often key regulators, and several TF families have been associated with distinct developmental and physiological processes (Riechmann *et al*, 2000). These enriched TF families may deserve further reverse genetic analysis, with a focus on boundary and AM formation. Lastly, we identified 180 gene-centered PDIs, between 103 TFs and 23 promoter regions, within a GRN for boundary and AM formation. Most of the PDIs are novel, and most TFs retrieved were heretofore uncharacterized. Further independent experimental analysis identified molecular phenotypes for 73.3% of the tested PDIs, suggesting that many of them are biologically relevant.

### Network architecture and regulatory genomic region hubs

Gene regulatory networks, like many other cellular networks, contain a small number of highly connected hubs, or nodes, and are characterized by a scale-free connectivity distribution (Barabasi & Oltvai, 2004). A previous gene-centered network analysis in worms identified TF interactor hubs (Barabasi & Oltvai, 2004); these TF interactor hubs connect to genes expressed in many cell types and are likely global regulators (Vermeirssen *et al*, 2007a). Although our study did not identify striking TF interactor hubs, we found clear, uneven distribution of PDIs associated with the tested promoter regions (Figs3B, C and 5B). In fact, the majority (53.9%) of PDIs were associated with one promoter region of each of two key regulators, *CUC2* and *LAS*. Our detailed analysis by independent experimental approaches showed that many of these PDIs are real (Figs4 and 6) and that mutation or over-expression of their upstream TF affects expression of their downstream target (Fig6) or even leads to AM and boundary phenotypes (Fig7). Our finding also reconciled the discrepancy that a conserved 3′ region is sufficient to direct *LAS* expression (Raatz *et al*, 2011), whereas an extended 5′ region is also able to define boundary-specific *LAS* expression (Goldshmidt *et al*, 2008). We found that CUC2, as a key regulator, can bind both the 5′ pLAS-12/13 and the 3′ regions B and C (Fig4B). Unfortunately, due to the high background introduced by region B, we were not able to test its PDIs by Y1H.

Previous GRN studies in yeast identified highly connected promoters (Yu *et al*, 2004a; Borneman *et al*, 2006), although no clear promoter hubs were identified in worms or *Arabidopsis* (Vermeirssen *et al*, 2007a; Brady *et al*, 2011). Also, a recent co-expression network analysis in *Arabidopsis* identified novel expression modules centered on *cis*-motifs (Ma *et al*, 2013), supporting the existence of promoter hubs. A notable feature of our Y1H analysis was the dissection of extended (up to 3.1 kb) promoter regions into short (180–320 bp) fragments, which not only provided better coverage of potential regulatory regions, but also ensured full transcriptional activation in yeast (Dobi & Winston, 2007). In fact, both putative promoter hubs were identified as more distant from the start codon, suggesting the need to study extended promoter regions. Nevertheless, the promoter dissection approach limited our study to a relatively small number of gene promoters. Further, larger-scale experiments would better evaluate the frequency and characteristics of promoter hubs.

By combining cell type-specific gene expression profiles and PDIs, we asked whether TFs and their targets are co-expressed and whether the regulations are regenerative or degenerative interactions. We found limited, but significant overlap in expression enrichment in boundary cells between TFs and their targets (Figs3B and 5B). A previous GRN analysis of the root stele reported similar observations (Brady *et al*, 2011), suggesting that TFs and their targets are not strictly co-expressed. By addition of inferred regulatory potential, we found that regenerative regulation involving either transcriptional activation or transcriptional repression represents the majority of PDIs from the small number of PDIs we studied in detail (Fig6C).

Our network analysis also highlighted high genetic redundancy of TFs. Although we were able to identify expression phenotypes at the molecular level for 73.3% of TFs tested (Fig6C and Supplementary Fig S4), we found morphological phenotypes for 31.8% of TFs tested. Because our selection of *Arabidopsis* lines for morphological phenotype characterization was influenced by availability of mutants and transgenic lines, as well as the literature, we expect the average percentage of observed phenotype to be lower than that. This observation is strikingly similar to the recent GRN study of root stele (Brady *et al*, 2011), implying high robustness of GRN.

## Materials and Methods

### Plant materials and generation of transgenic plants

The *Arabidopsis thaliana* accession Columbia (Col-0) was used as the wild-type unless otherwise specified. TRAP-seq lines were in the Landsberg *erecta* (L*er*) background. Information on the detailed genetic background of mutants and transgenic lines used in this study is provided in Supplementary Table S11. Plants were grown in the greenhouse on soil at 22°C. Plants used for TRAP-seq experiments were grown under constant illumination, plants used for AM phenotypic characterization were grown under short-day conditions (8 h light/16 h dark) for 28 days before moving to long-day conditions (16 h light/8 h dark), and all other plants were grown under long-day conditions.

To obtain *pLAS>>HF:RPL18* and *pAS1>>HF:RPL18* lines, cell type-specific *pLAS::LhG4* (Goldshmidt *et al*, 2008) and *pAS1::LhG4* (Eshed *et al*, 2001) drivers were crossed into a *pOp::HF-RPL18* driver line that also contains a linked *pOp::GUS* (Jiao & Meyerowitz, 2010), all in the L*er* background.

The *p35S::DRN-GR* was made by inserting the *DRN* coding sequence amplified from cDNA in-frame upstream of the GR coding sequence in the pGREEN0229-35S::GR vector (Yu *et al*, 2004b). For constructing *pRPS5A::CUC2-GR-HA* and *pRPS5A::RAX1-GR-HA*, a 1.7-kb fragment upstream of the ubiquitously expressed *RPS5A* coding region (Weijers *et al*, 2001) was amplified and inserted into BJ36. The *Arabidopsis CUC2* or *RAX1* cDNA was cloned downstream of the *RPS5A* promoter with GR and HA sequences. The construct was then transferred into the binary vector pMOA34. All binary constructs were transformed into Col-0. Transgenic lines with a reproducible phenotype after Dex treatment were selected and used for subsequent analysis. Dex and cycloheximide treatments were performed as previously described (Han *et al*, 2014).

### TRAP-seq

Seedlings grown on 1/2 MS agar plates containing 1% sucrose were used at 7 DAG. Shoots were frozen in liquid nitrogen, and isolation of polysomes and affinity purification of HF-RPL18-containing polysomes using anti-FLAG beads were carried out as previously described (Jiao & Meyerowitz, 2010; Wang & Jiao, 2014). Total RNA and subsequent poly(A)^+^ RNA were isolated from each replicate and subjected to RNA-seq library preparation as described (Jiao & Meyerowitz, 2010; He & Jiao, 2014). Libraries were sequenced as 50-mers using HiSeq2000 (Illumina, San Diego, CA, USA) with standard settings. Three independent biological replicates were included for each cell type.

### Read mapping and quantification of expression

Reads were mapped to the *Arabidopsis* Information Resource TAIR10 reference genome build with TopHat2 (version 2.0.9) and BOWTIE (version 2.1.0) allowing up to two mismatches (Kim *et al*, 2013) after filtering the low-quality reads (PHRED quality score < 20). The gene locus expression levels were calculated based on mapping outputs after removing reads mapped to rRNAs and tRNAs using Cuffdiff2 (version 2.1.1) (Trapnell *et al*, 2013), and expression levels were normalized to the RPKM unit using edgeR (Robinson *et al*, 2010) with significant expression cutoff value set to RPKM > 0.5 (Jiao & Meyerowitz, 2010). Differential expression was assessed with edgeR, and the cutoff value was > twofold change in expression with Benjamini–Hochberg adjusted *P *<* *0.001.

### Gene ontology, enrichment, and promoter motif analysis

Gene ontology term enrichment analysis was performed using agriGO with the singular enrichment analysis method (Du *et al*, 2010). Lists of the phytohormone-responsive genes were obtained from Jiao and Meyerowitz (2010). The cytokinin-responsive gene list was updated to include more comprehensive results from a recent study (Bhargava *et al*, 2013). TF classification was based on databases of AGRIS, PlantTFDB, and RARTF (Iida *et al*, 2005; Palaniswamy *et al*, 2006; Guo *et al*, 2008). Lists of TFs and hormone-responsive genes are available in Supplementary Tables S6 and S7. The gene enrichment analysis was quantified by log odds ratio (LR) as previously described (Jiao & Meyerowitz, 2010). Hypergeometric distribution was used to assess the statistical significance (*P*-value) of the enrichment of promoter hubs. Kernel density curves were employed to examine gene abundance according to their log_2_FC value in the translatomes. The TFs for Y1H screening and TFs in PDIs are listed in Supplementary Tables S8 and S10.

Promoter motif enrichment was analyzed as previously described (Jiao *et al*, 2005; Jiao & Meyerowitz, 2010). The genome sequences 2 kb upstream from annotated translation start sites for boundary-specific or leaf-specific genes were retrieved from the TAIR10 genome build to identify over-represented known sequence motifs using an enumerative approach with Elefinder (http://stan.cropsci.uiuc.edu/tools.php). Those elements meeting an expected (*E*) value smaller than 10^−4^ were selected for further comparison.

### Construction of Y1H bait strains

Yeast (*Saccharomyces cerevisiae*) strain Y1HGOLD (MAT α) was used as the donor strain to express the TF library containing fusion proteins of GAL4-AD-TF. The components of yeast complete medium and different synthetic drop-out (SD) media were obtained from Clontech and prepared according to the manufacturer's instructions.

Promoter fragments of *CUC2*, *LAS*, *MiR164c,* and *STM* were amplified from genomic DNA using specific primers (Supplementary Table S12). The fragments were verified by sequencing and cloned into pAbAi (Clontech, Mountain View, CA, USA). All the bait plasmids were linearized by *Bst*BI and were integrated into yeast strain Y1HGOLD using PEG-mediated transformation according to the user manual (*Yeast Hand Book*; Clontech, PT3024-1). Transformants were selected on media lacking uracil, verified by PCR using a promoter-specific primer and a yeast chromosome primer (Supplementary Table S12), and tested for auto-activation according to the manufacturer's instructions.

### Construction of AD-TF prey clones

All AD-TF prey clones are derived from pDEST22 (Life Technologies, Carlsbad, CA, USA; Ou *et al*, 2011), and Gateway cloning was used to generate additional AD-TF clones. The cDNA clones were either from ABRC or cloned in this work, both using the pENTR/D-TOPO vector (Life Technologies). To generate Gal4-AD-TF constructs, Gateway LR recombination reactions were performed between pENTR/D-TOPO-TFs and pDEST22 to obtain pDEST22-TF.

### Transformation-based Y1H screening

A direct, transformation-based assay was used following published protocols, unless otherwise specified (Mitsuda *et al*, 2010; Brady *et al*, 2011). Briefly, AD-TF plasmids from the TF prey library were directly transformed into Y1HGOLD bait strains harboring genomic promoter-reporters, and transformants were selected on media lacking uracil and tryptophan but containing 800 ng/ml aureobasidin A (AbA). An equal amount of transformed yeast culture was plated on medium lacking uracil and tryptophan without addition of AbA to control for transformation efficiency. We used a limited pooling strategy by mixing equal amounts of four AD-TF plasmids for each transformation. Positive interactions were identified based on growth ability after transformation, on AbA-containing medium for 3 days, according to the manufacturer's manual. For each pool containing a positive interaction, the four AD-TFs of this pool were individually transformed and screened to identify the AD-TF(s) involved in the positive interaction. All interactions were validated by retesting using the same procedure.

### RT-PCR and quantitative real-time PCR

Total RNA from inflorescences of four plants at 8 days after bolting was extracted using the AxyPrep Multisource RNA MiniPrep kit (Axygen, Tewksbury, MA, USA). First-strand cDNA was synthesized with 2 μg total RNA by TransScript One-step gDNA Removal and cDNA synthesis SuperMix (TransGen, Beijing, China) using anchored oligo-dT primers according to the manufacturer's instructions. Quantitative real-time PCR (qRT–PCR) was performed on a Bio-Rad CFX96 real-time PCR detection system using KAPA SYBR FAST qPCR kit (KAPA Biosystems, Beijing, China). *TUB6* (AT5G12250) was chosen to normalize the relative expression as it has been shown to be a superior reference gene for qRT–PCR analysis (Han *et al*, 2014). Gene-specific primers (Supplementary Table S12) were used to amplify each gene, and two independent biological experiments, each run in triplicate, were applied for each mutant or transgenic plant.

### Modeling

For each putative PDI, we used weighted least squares regression to model the relationship between the expression of the TF and its target gene in both wild-type and mutant plants, as described before (Brady *et al*, 2011). The slope of the line can suggest the activation or repression activity of a TF. Its steepness can also provide an estimate of the strength of the TF acting on its target. The *P*-value of the line represents the probability of whether the expression of the two TFs can result in a regression line.

### Electrophoretic mobility shift assay

Fusion proteins were produced in prokaryotic expression systems. The DNA-binding domain of ARR1, ARRM (236aa–299aa) (Taniguchi *et al*, 2007), SPL9-binding domain (64aa–153aa) (Liang *et al*, 2008), and SPL15-binding domain (49aa–138aa) (Liang *et al*, 2008) were amplified by gene-specific primers (Supplementary Table S12). The coding sequences were ligated to the vector pGEX-6P-1, and proteins were successfully expressed with the GST tag. GST-fused proteins were purified using glutathione-Sepharose 4B, as described before (Tian *et al*, 2008). Amplified full-length protein coding sequence of CUC2 was cloned into the pETMALc vector to fuse with the MBP tag (Pryor & Leiting, 1997). Expressed MBP-CUC2 protein was purified by amylase resin (NEB, Ipswich, MA, USA) according to the manufacturer's instructions. Protein concentration was measured by Bradford protein assay kit (GenStar, Beijing, China).

Biotin-labeled primers (sequences in Supplementary Table S12) were synthesized by Sangon Biotech (Shanghai, China). Probes were amplified using labeled primers, and corresponding competitors were amplified using primers of the same sequences without labeling. Binding reactions were performed in a 15-μl volume containing 50 ng protein and 20 fmol labeled DNA fragment using the Pierce LightShift Chemiluminescent EMSA Kit (Thermo Fisher, Rockford, IL, USA). Competition experiments were performed by adding 100- to 200-fold unlabeled DNA. The incubated mixture was separated in a 5% native polyacrylamide gel in 0.5× TBE at room temperature and then transferred to positively charged nylon membrane. After cross-linking under UV light, binding reactions were detected following the manufacturer's instructions.

### Chromatin immunoprecipitation

Ten-day-old seedlings or inflorescences of approximately 4 week old in *pRPS5A::CUC2-GR-HA* were induced with Dex as described above. Seedlings or inflorescence material (∼800 mg) from Dex-treated and mock-treated plants were harvested 2 h after treatment and fixed with 1% (v/v) formaldehyde under vacuum for 10 min (Han *et al*, 2014). Chromatin was sheared to an average size of 1,000 bp by sonication after nuclei were isolated and lysed. Immunoprecipitations were performed with or without anti-HA (Beyotime, Nantong, China). The precipitated DNA was isolated and purified to use as a template for amplification of promoter sequences with primers described in Supplementary Table S12. Two independent sets of biological samples were used.

### Accession number

NCBI Short Read Archive SRP042272.

## Supporting Information

Supplementary Tables

Review Process File

Source Data for Figure 3C

Source Data for Figure 4B

Source Data for Figure 4E
