# Supplementary material for: An organ boundary-enriched gene regulatory network uncovers regulatory hierarchies underlying axillary meristem initiation
Source: Mol Syst Biol. 2014 Oct 30;10(10):1–2. doi: 10.15252/msb.20145470 (PMC4299377; doi:10.15252/msb.20145470)
Supplement: Supplementary file 1 [file msb0010-0755-sd1.pdf]

**Figure S1**. (A) Expression of *pLAS>>GFP-ER* (green), and (B) pattern of *AS1* promoter driven GUS expression in longitudinal sections through vegetative shoot apex of 25-day-old wild-type like plants showing cell type specific expression promoter activities. Bars = 50 μm

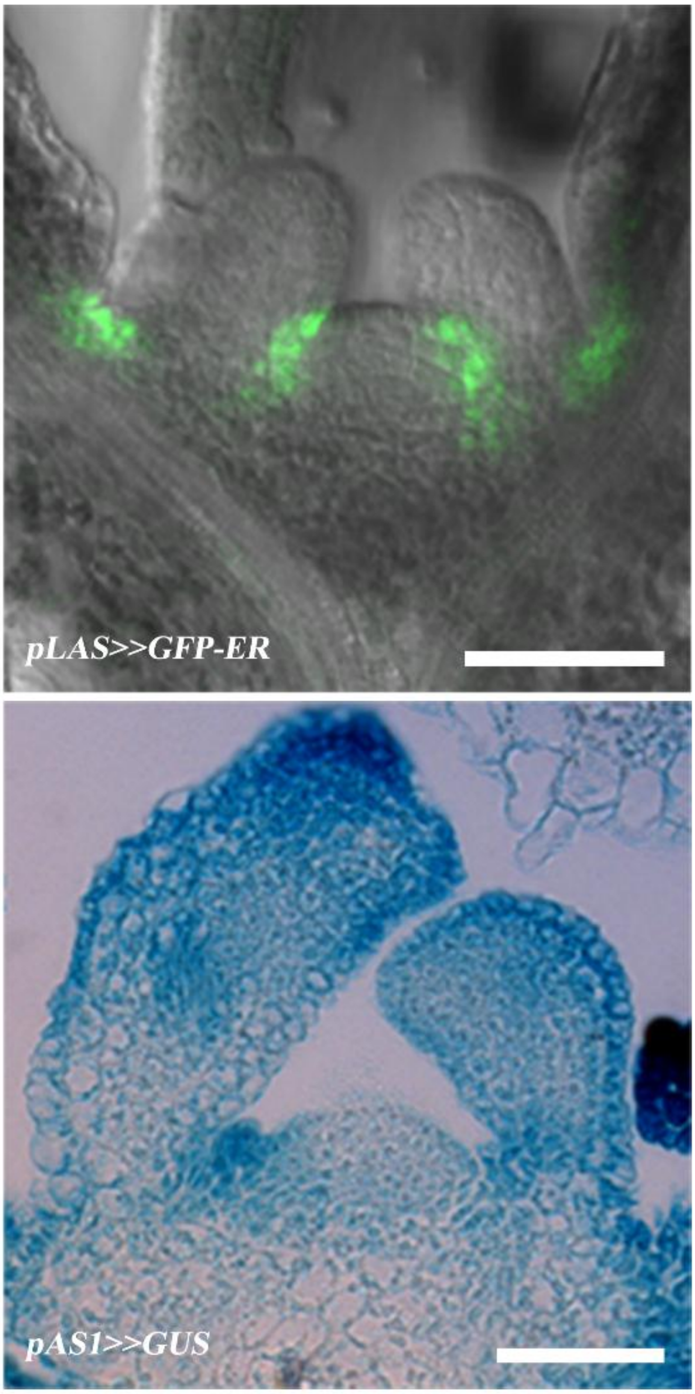

**Figure S2**. GO analysis identified significantly overrepresented (FDR adjusted *P* < 0.01) gene categories for the leaf cell-specific transcripts. Color bar: significance levels for categories by hypergeometric test with FDR correction.

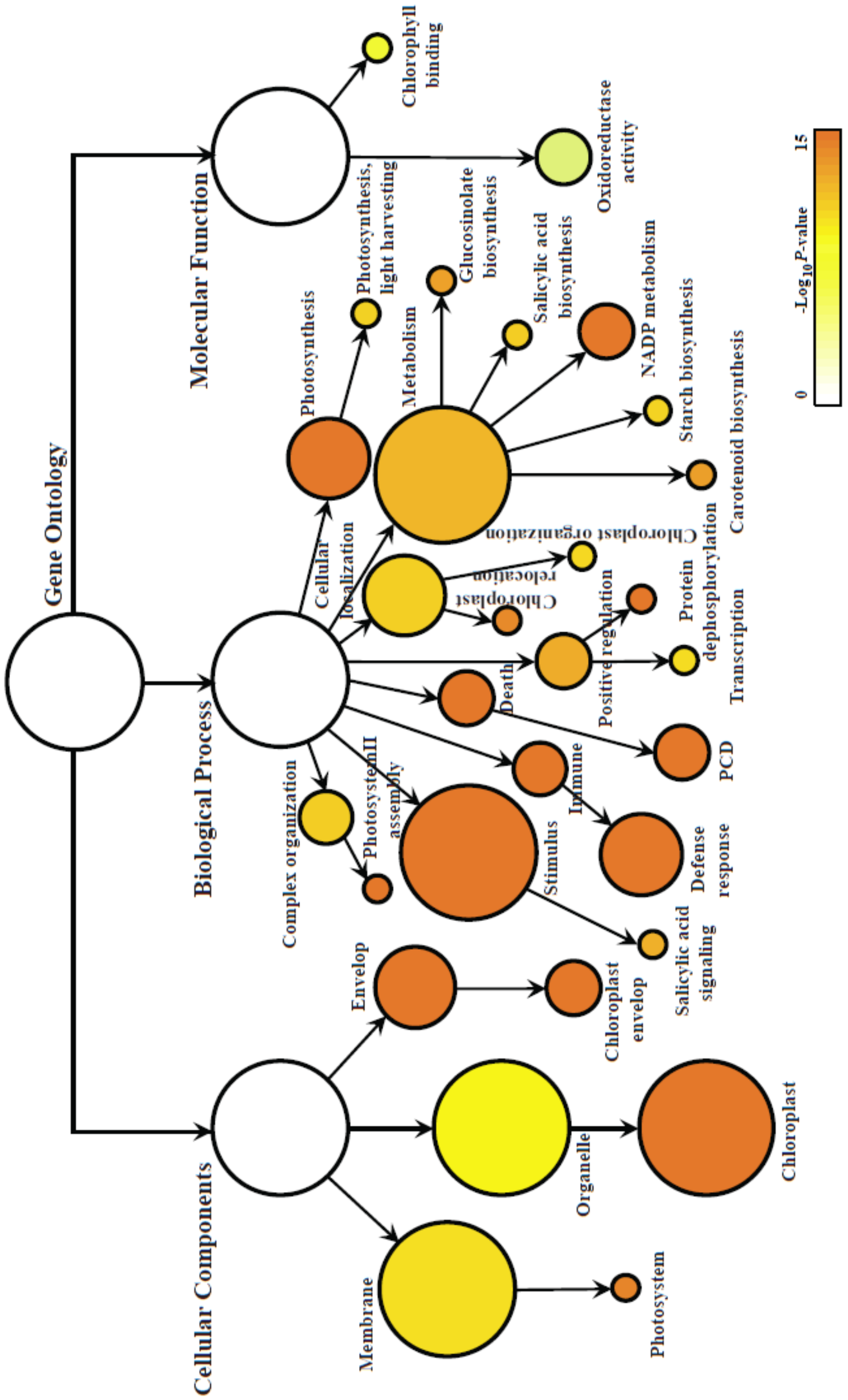

**Figure S3**. Reporter assay procedure and readout with a promoter fragment and 96 TF pools w/ and w/o AbA selection. Each TF pool used in the first round included four TFs, and each TF in positive pools was individually tested in the second-round screen.

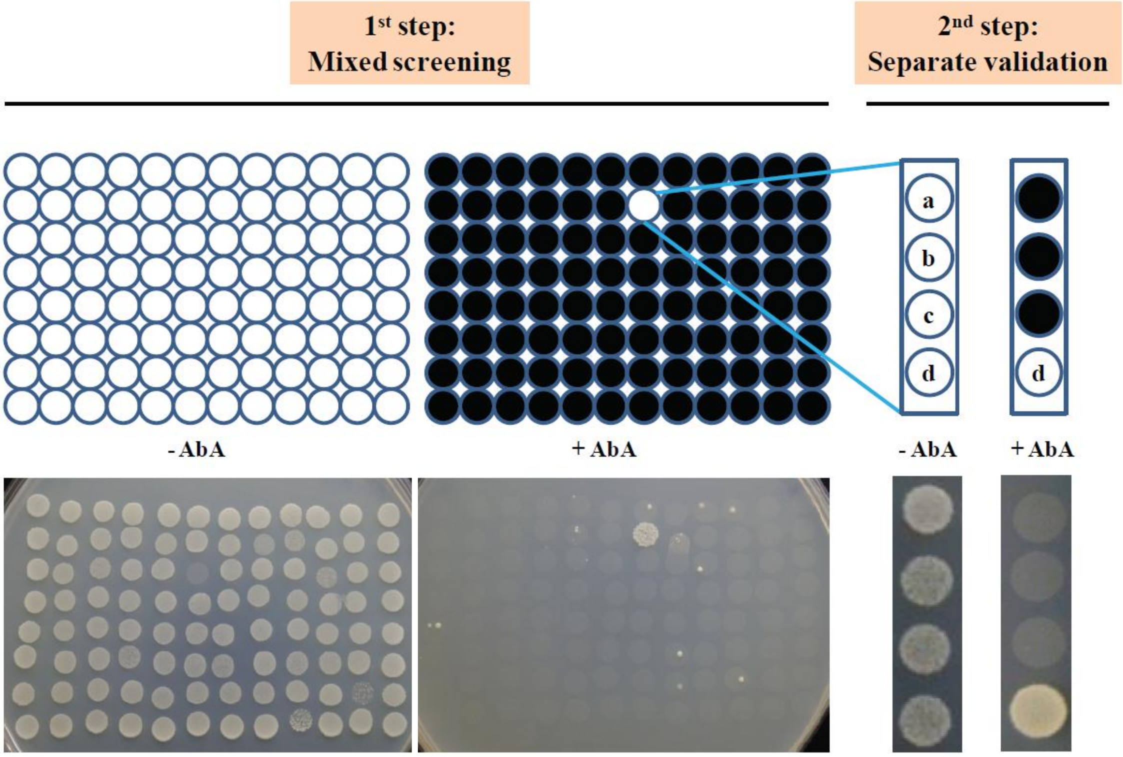

**Figure S4**. Weighted least square regression (WLSR) plots. WLSR was used to predict the potential of regulatory relationships between a target gene and its upstream TF. We used WLSR to fit a line that incorporates variance measurements across three technical replicates for each of the two biological replicates (six replicates in total). The slope of a line predicts the degree of activation or repression with a *P* value represents the confidence level for the regulation. See *Materials and Methods* for more details.

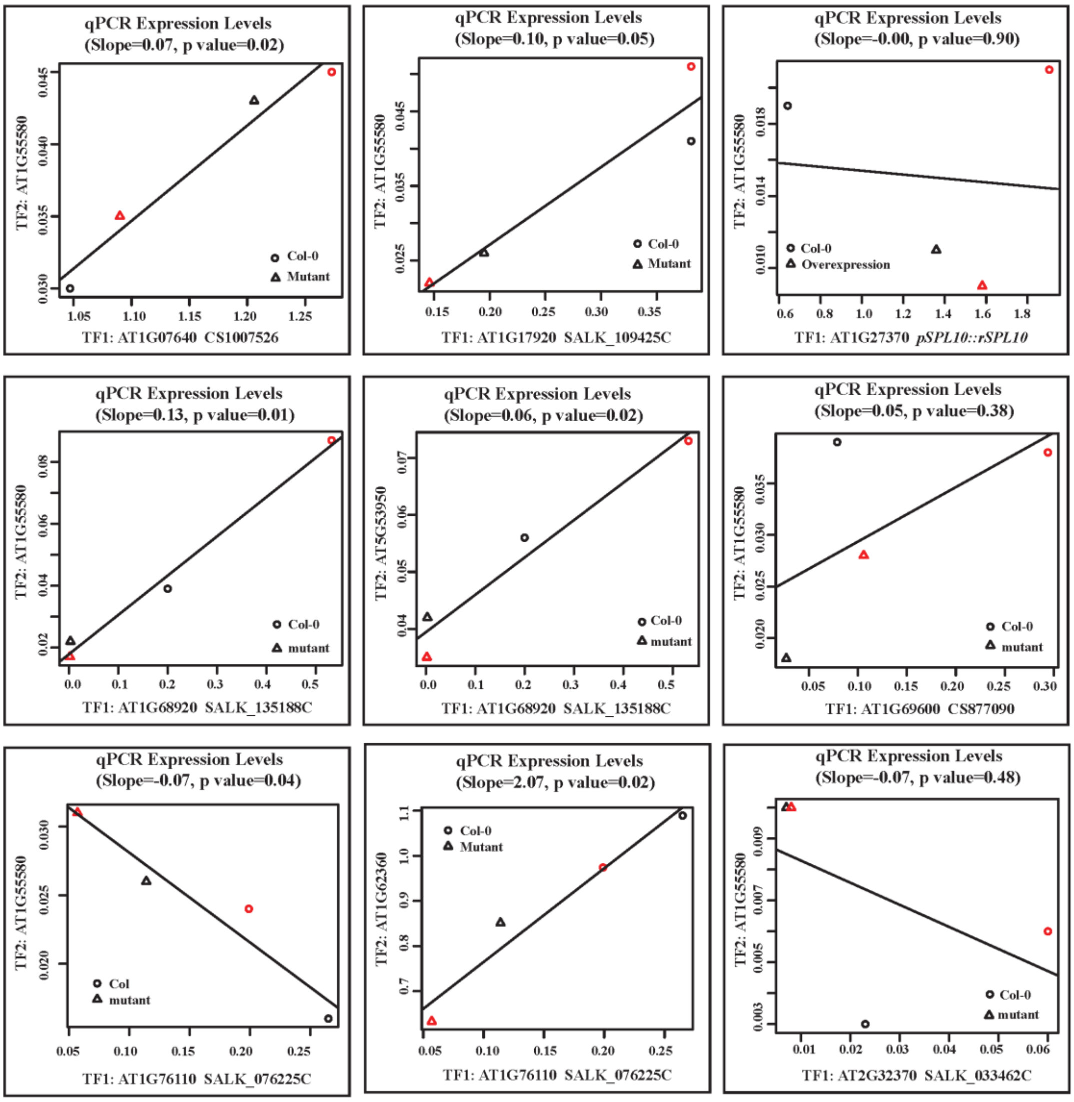

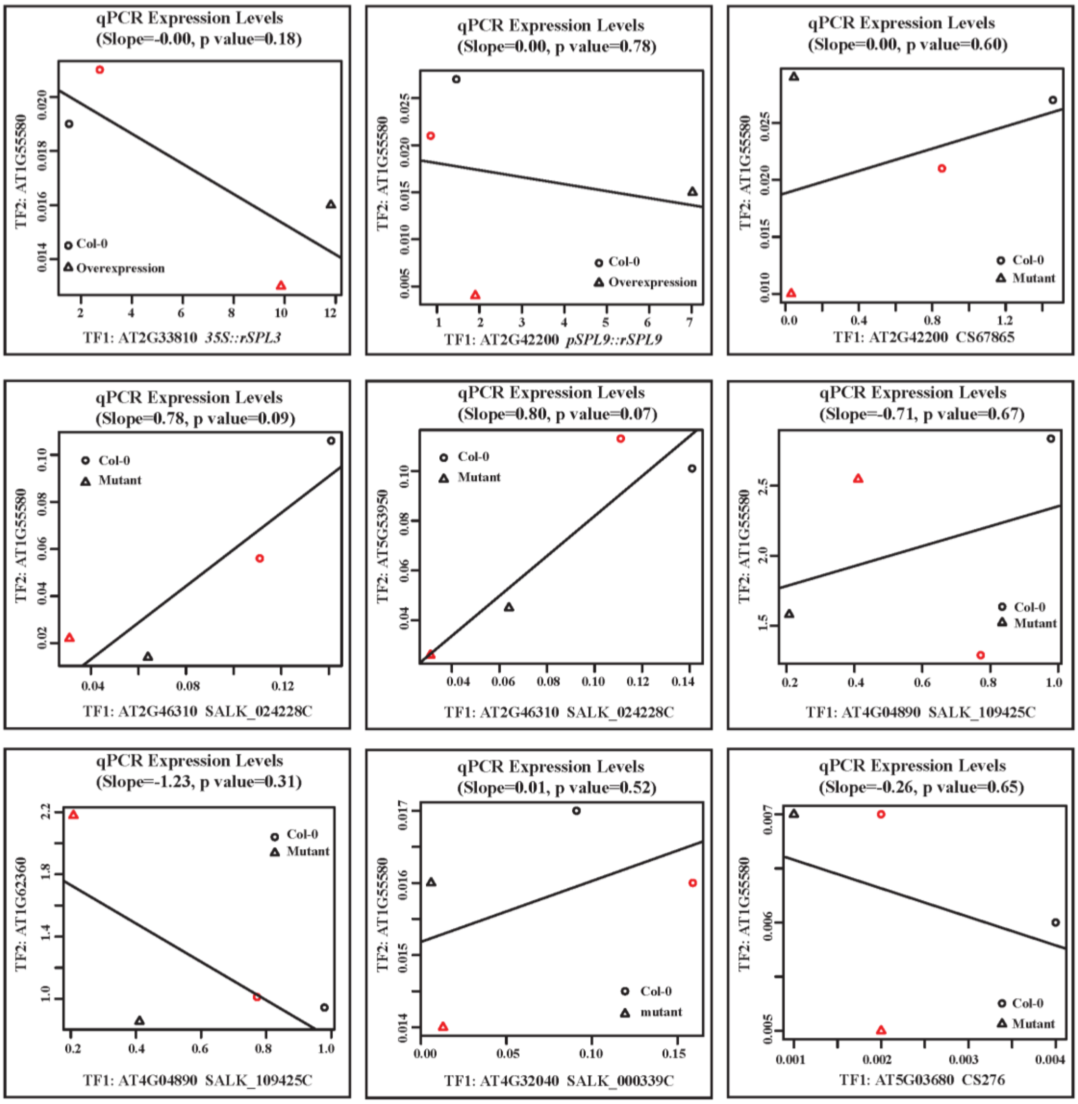

qPCR Expression Levels
(Slope=-0.00, p value=0.18)
TF2: AT1G55580
Col-0
Overexpression
TF1: AT2G33810 35S::rSPL3
qPCR Expression Levels
(Slope=0.00, p value=0.78)
TF2: AT1G55580
Col-0
Overexpression
TF1: AT2G42200 pSPL9::rSPL9
qPCR Expression Levels
(Slope=0.00, p value=0.60)
TF2: AT1G55580
Col-0
Mutant
TF1: AT2G42200 CS67865
qPCR Expression Levels
(Slope=0.78, p value=0.09)
TF2: AT1G55580
Col-0
Mutant
TF1: AT2G46310 SALK_024228C
qPCR Expression Levels
(Slope=0.80, p value=0.07)
TF2: AT5G53950
Col-0
Mutant
TF1: AT2G46310 SALK_024228C
qPCR Expression Levels
(Slope=-0.71, p value=0.67)
TF2: AT1G55580
Col-0
Mutant
TF1: AT4G04890 SALK_109425C
qPCR Expression Levels
(Slope=-1.23, p value=0.31)
TF2: AT1G62360
Col-0
Mutant
TF1: AT4G04890 SALK_109425C
qPCR Expression Levels
(Slope=0.01, p value=0.52)
TF2: AT1G55580
Col-0
mutant
TF1: AT4G32040 SALK_000339C
qPCR Expression Levels
(Slope=-0.26, p value=0.65)
TF2: AT1G55580
Col-0
Mutant
TF1: AT5G03680 CS276

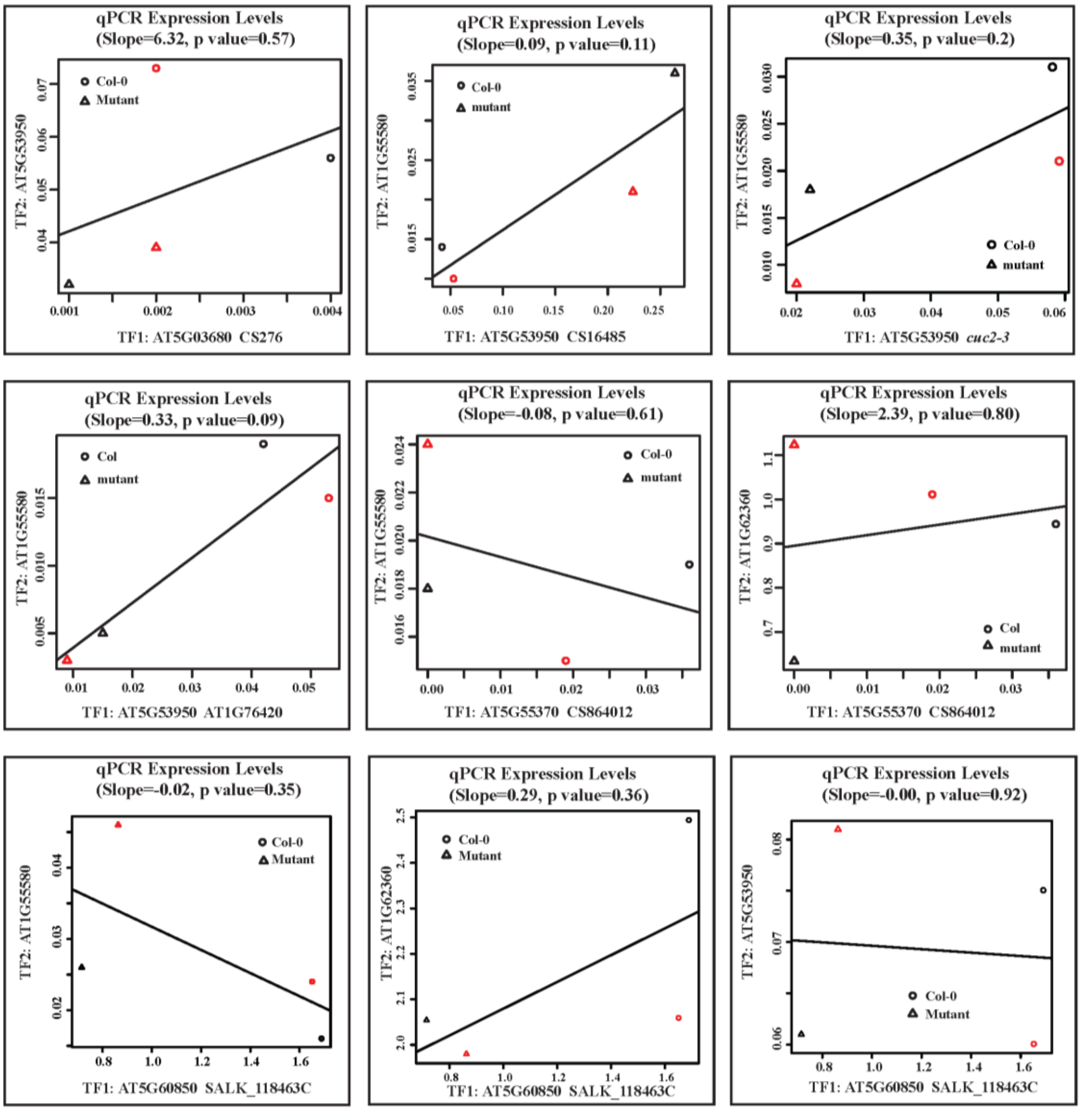

qPCR Expression Levels
(Slope=6.32, p value=0.57)
Col-0
Mutant
TF2: AT5G53950
TF1: AT5G03680 CS276
qPCR Expression Levels
(Slope=0.09, p value=0.11)
Col-0
mutant
TF2: AT1G55580
TF1: AT5G53950 CS16485
qPCR Expression Levels
(Slope=0.35, p value=0.2)
Col-0
mutant
TF2: AT1G55580
TF1: AT5G53950 cuc2-3
qPCR Expression Levels
(Slope=0.33, p value=0.09)
Col
mutant
TF2: AT1G55580
TF1: AT5G53950 AT1G76420
qPCR Expression Levels
(Slope=-0.08, p value=0.61)
Col-0
mutant
TF2: AT1G55580
TF1: AT5G55370 CS864012
qPCR Expression Levels
(Slope=2.39, p value=0.80)
Col
mutant
TF2: AT1G62360
TF1: AT5G55370 CS864012
qPCR Expression Levels
(Slope=-0.02, p value=0.35)
Col-0
Mutant
TF2: AT1G55580
TF1: AT5G60850 SALK_118463C
qPCR Expression Levels
(Slope=0.29, p value=0.36)
Col-0
Mutant
TF2: AT1G62360
TF1: AT5G60850 SALK_118463C
qPCR Expression Levels
(Slope=-0.00, p value=0.92)
Col-0
Mutant
TF2: AT5G53950
TF1: AT5G60850 SALK_118463C

**Figure S5**. Interactions between TFs and their targets indicated by WLSR analysis. Regulatory interaction strength is shown by arrow and edge width. The opacity represents the interaction *P* value, in which darker arrow or edge opacity indicates a more-significant interaction.

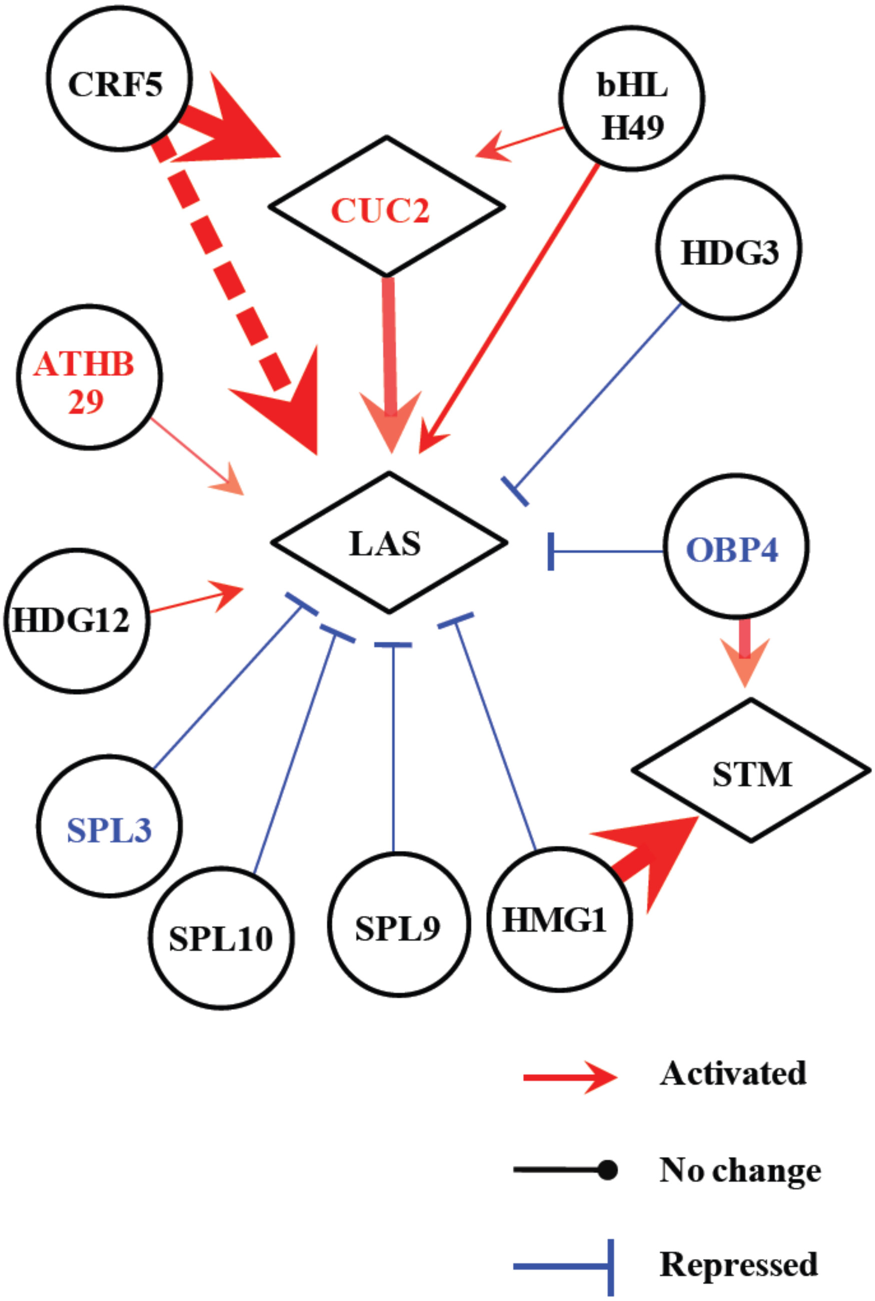

**Figure S6**. Phenotypic characterization of new mutants affecting leaf margin development and cotyledon formation. (**A**) Leaf series of Col-0, *cuc2-1D*, *ptl-1* and *hmg1*. Leaves with enhanced margin serration are highlighted by arrows. (**B**) Cotyledon morphology of Col-0 and *hdg12*. Tricots and fused cotyledons in the *hdg12* mutant are shown. (**C**) Disordered leaf phyllotaxy and fusion phenotypes of *hdg12*.(**D**) Phenotype statistics of *hdg12*.

A

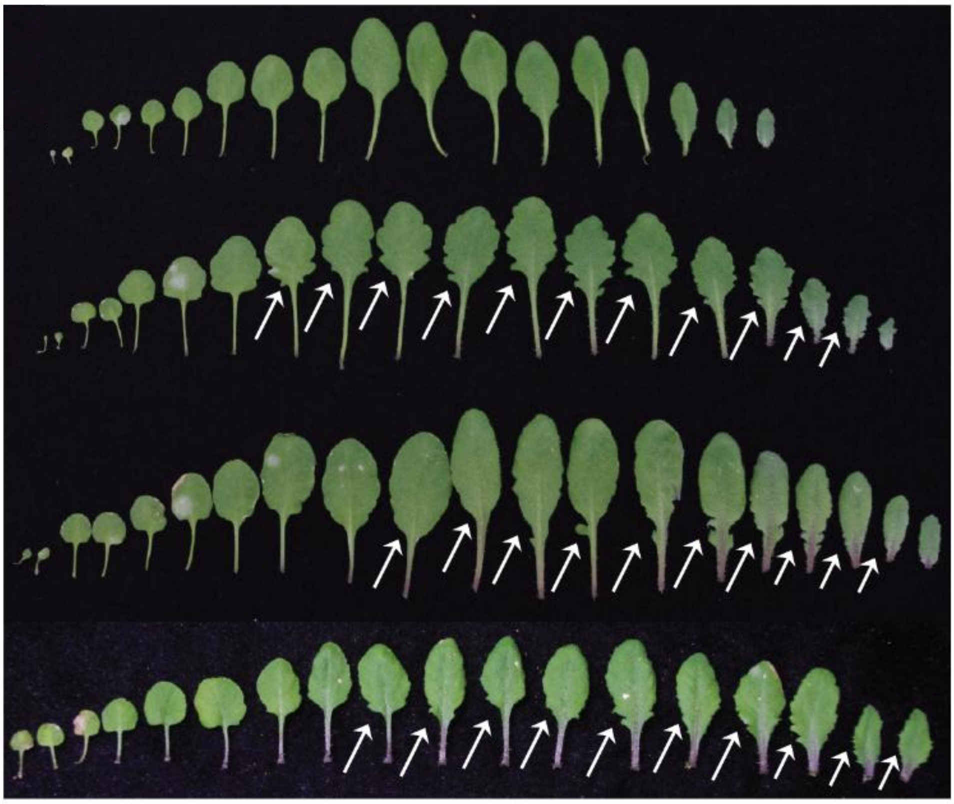


B

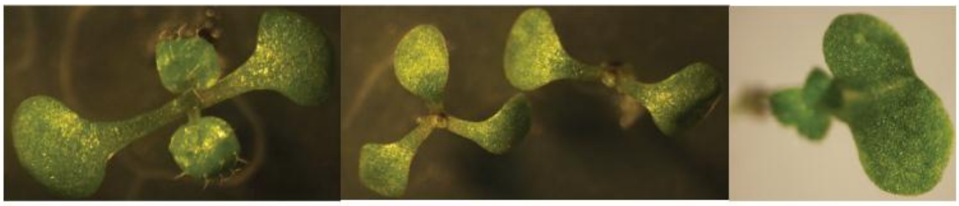


C

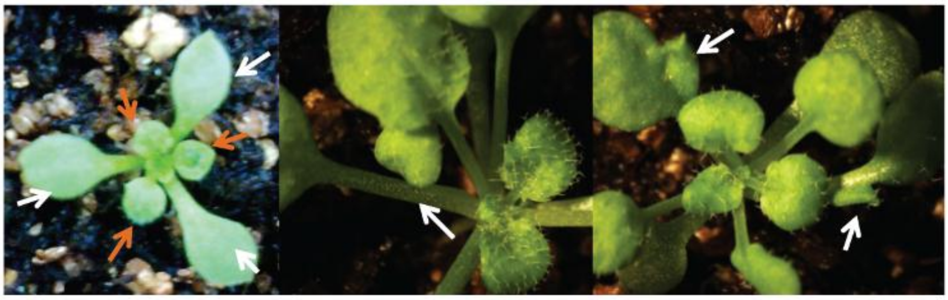


D

| | Three cotyledons | Fused cotyledons | Delayed development |
|---|---|---|---|
| Col-0 | 0/120 (0%) | 0/120 (0%) | 0/120 (0%) |
| *hdg12* | 13/248(5.2%) | 13/248 (2.20%) | 248/248 (100%) |
