# Supplementary figures and images for: An organ boundary-enriched gene regulatory network uncovers regulatory hierarchies underlying axillary meristem initiation
Source: Mol Syst Biol. 2014 Oct 30;10(10):1–2. doi: 10.15252/msb.20145470 (PMC4299377; doi:10.15252/msb.20145470)

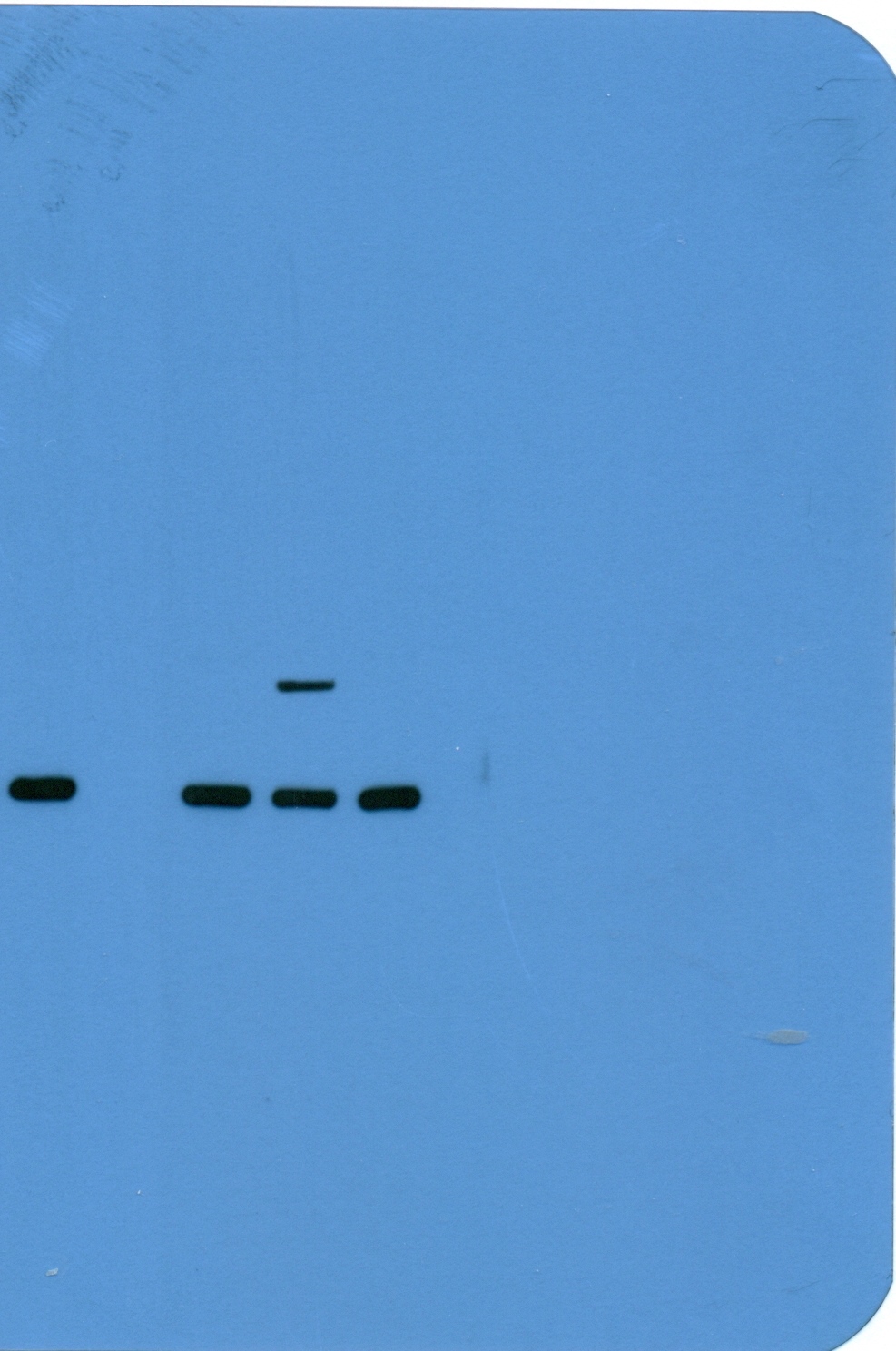

Supplement: Supplementary file 5 [file msb0010-0755-sd5.zip › Source Data for Figure 4B/ARR1_pLAS-12.jpg]

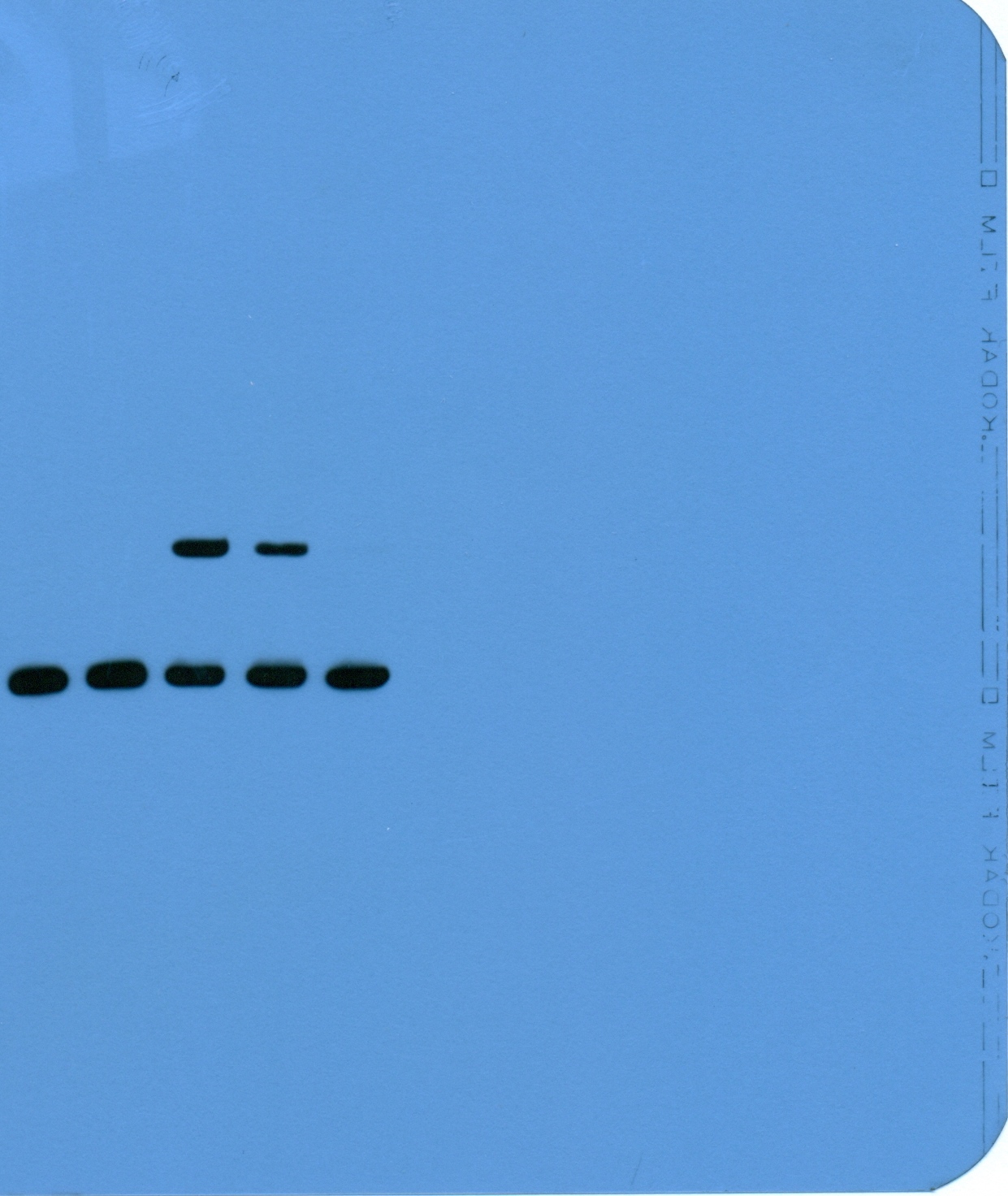

Supplement: Supplementary file 5 [file msb0010-0755-sd5.zip › Source Data for Figure 4B/ARR1_pLAS-13.jpg]

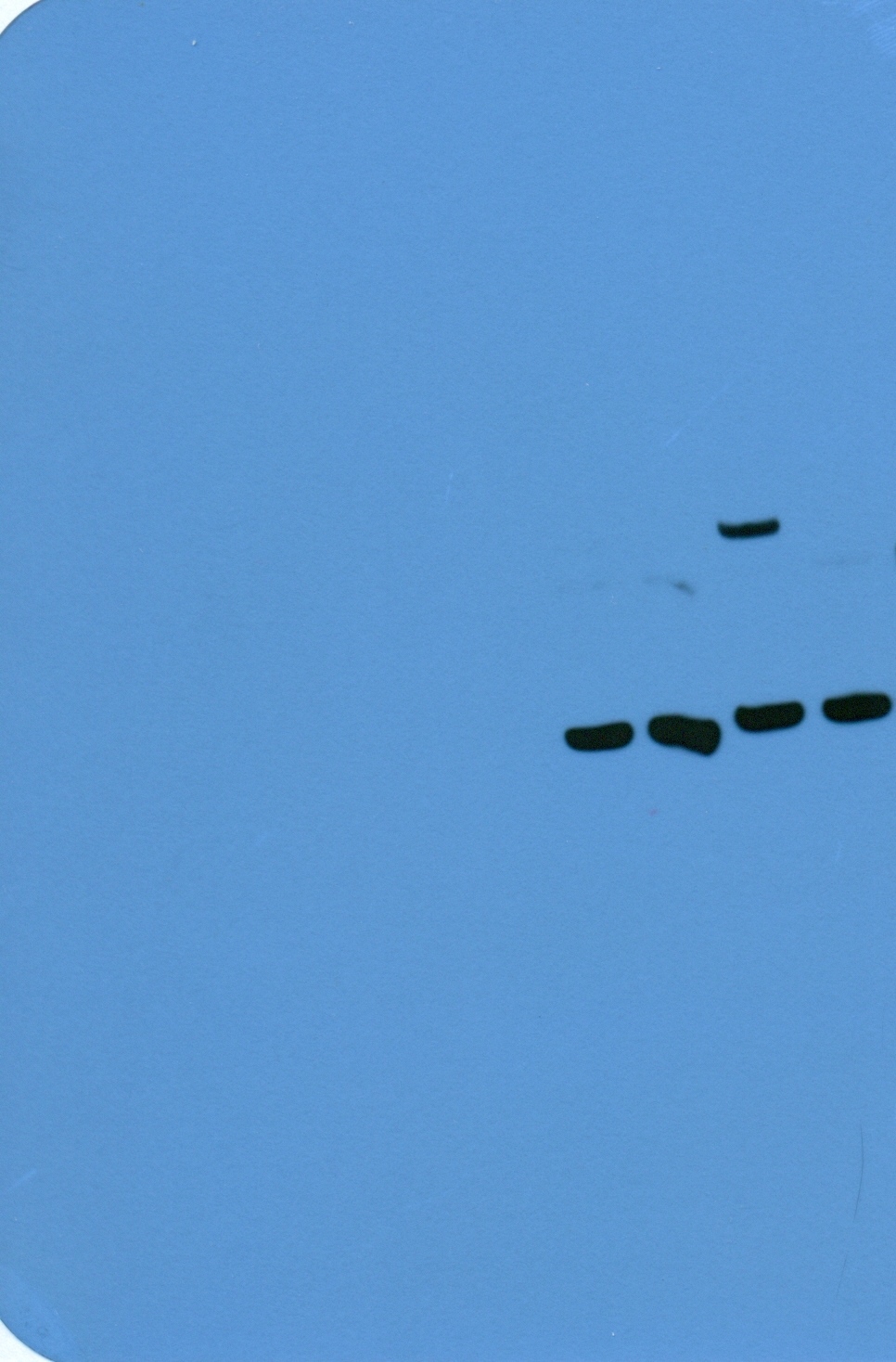

Supplement: Supplementary file 5 [file msb0010-0755-sd5.zip › Source Data for Figure 4B/CUC2_pLAS-12.jpg]

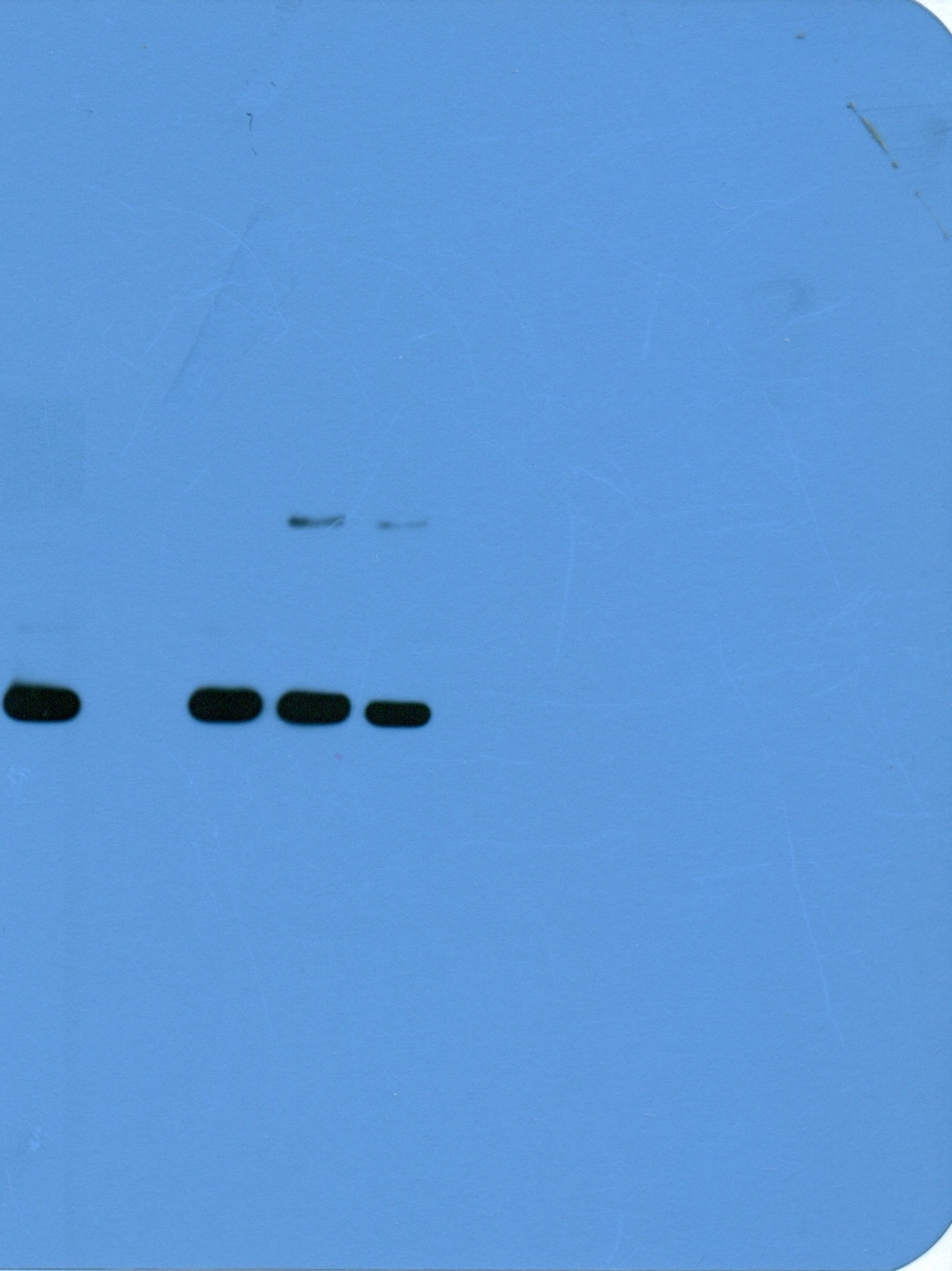

Supplement: Supplementary file 5 [file msb0010-0755-sd5.zip › Source Data for Figure 4B/CUC2_pLAS-13.jpg]

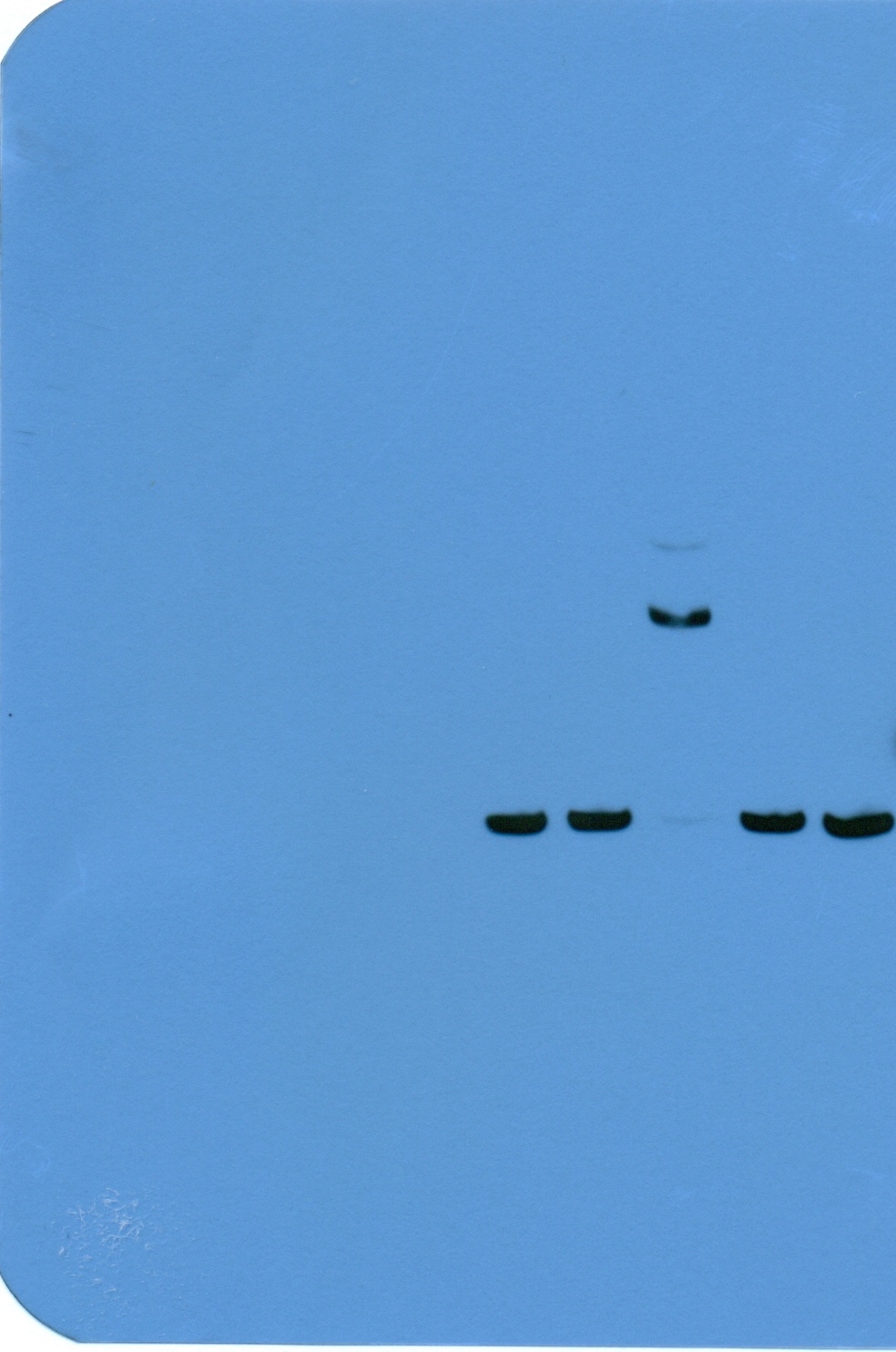

Supplement: Supplementary file 5 [file msb0010-0755-sd5.zip › Source Data for Figure 4B/CUC2_pMiR164c-5.jpg]

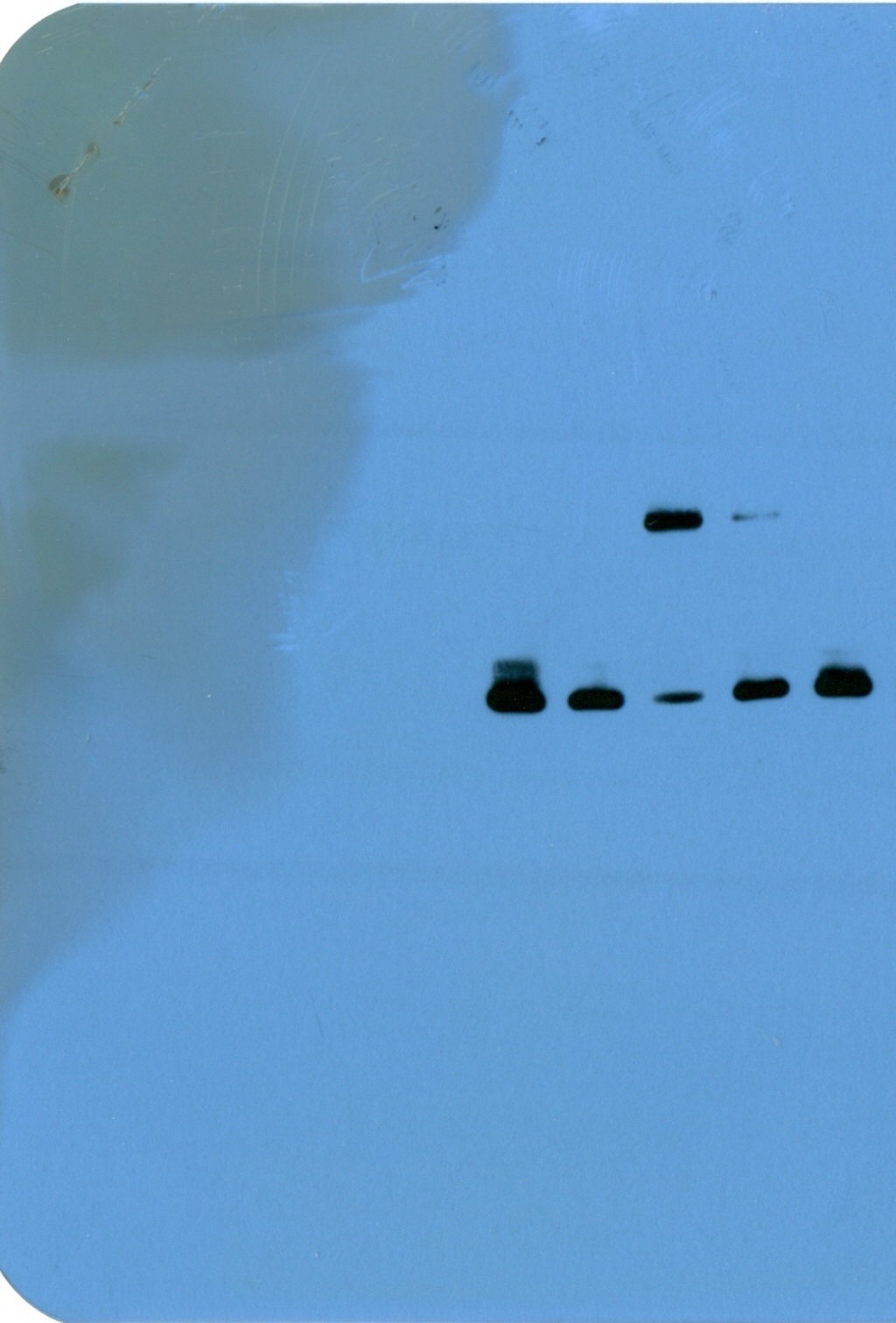

Supplement: Supplementary file 5 [file msb0010-0755-sd5.zip › Source Data for Figure 4B/SPL9_pLAS-12.jpg]

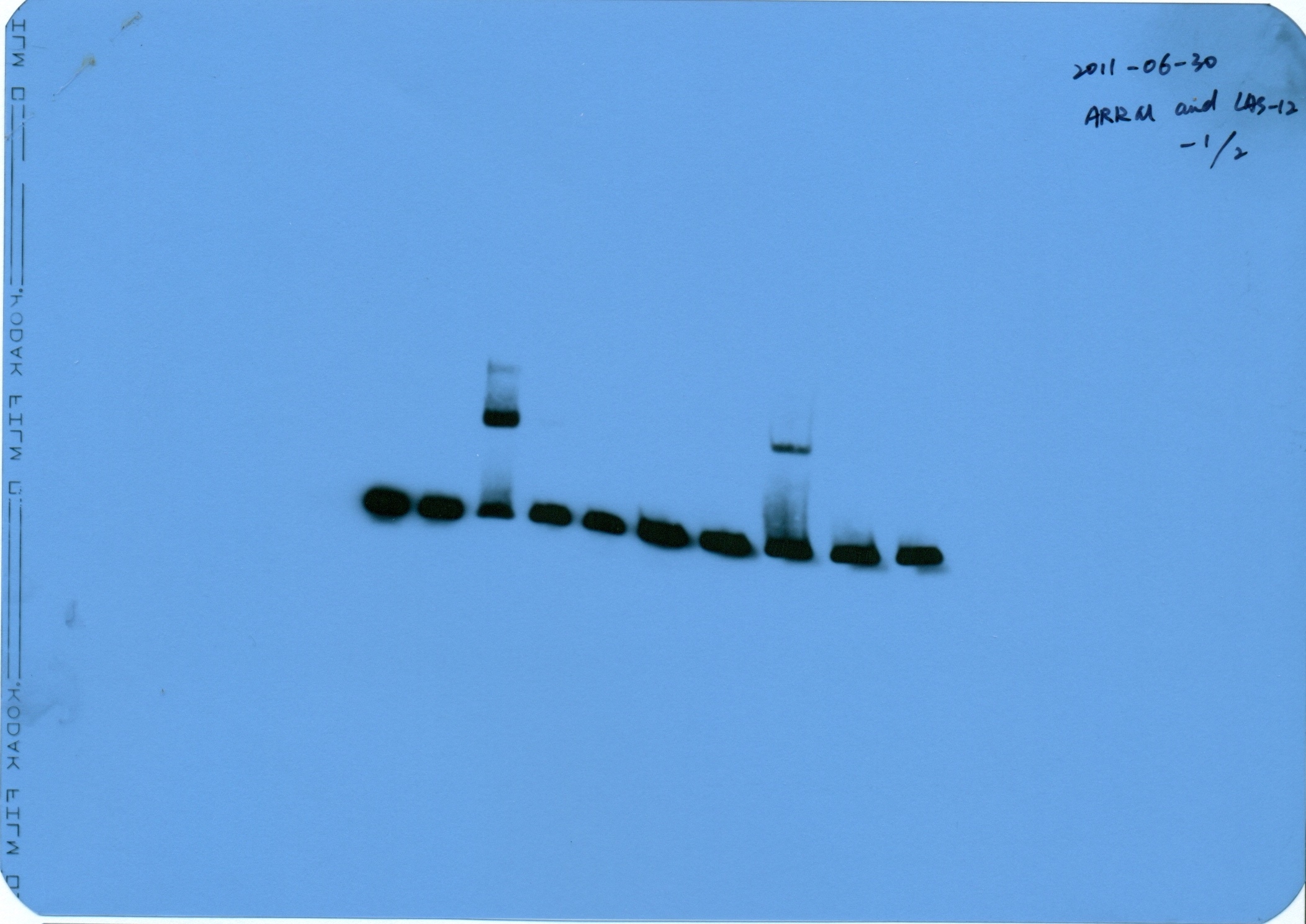

Supplement: Supplementary file 6 [file msb0010-0755-sd6.zip › Sources Data for Figure 4E/ARR1_pLAS-12-1_and_2.jpg]

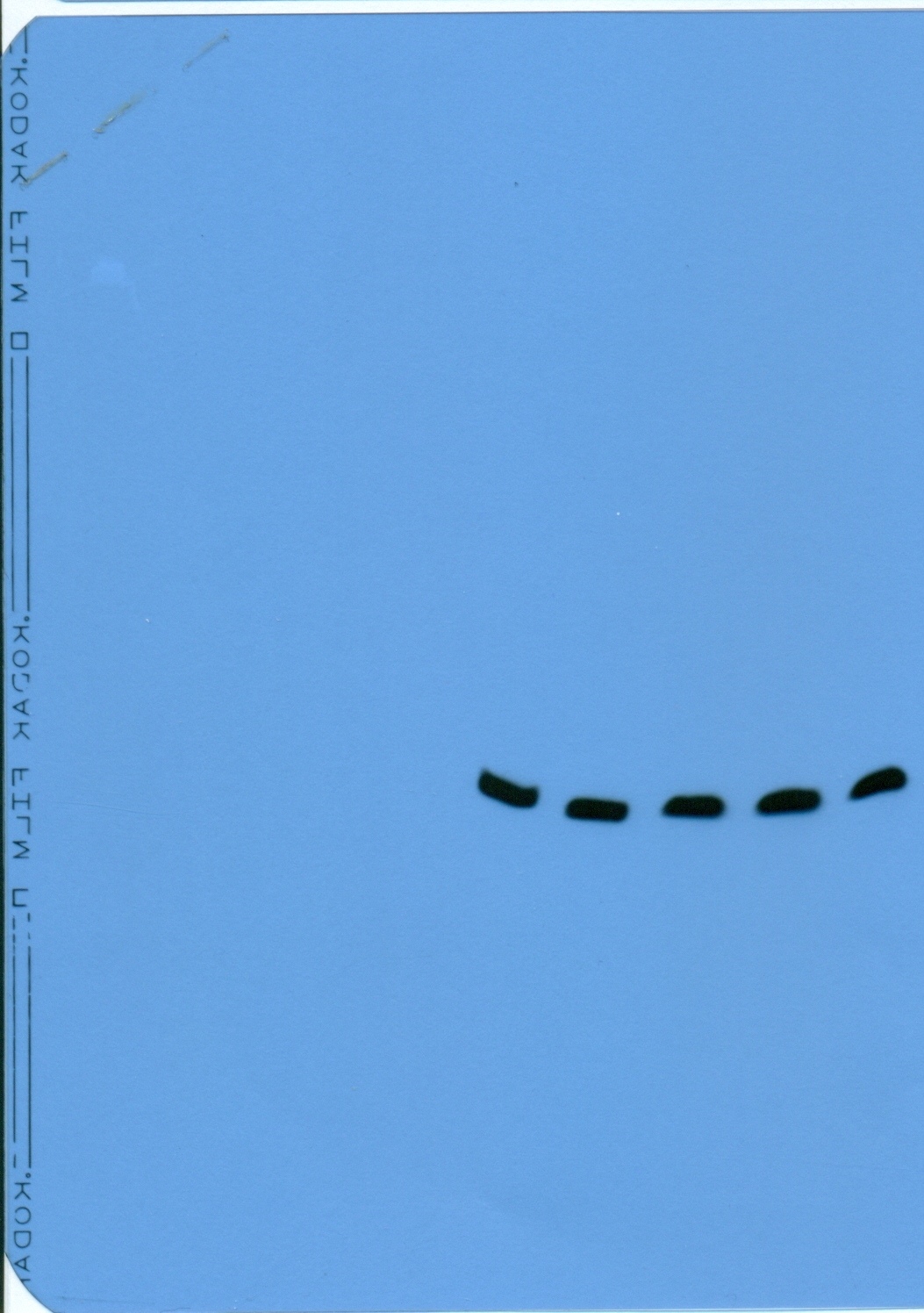

Supplement: Supplementary file 6 [file msb0010-0755-sd6.zip › Sources Data for Figure 4E/ARR1_pLAS-12-3.jpg]

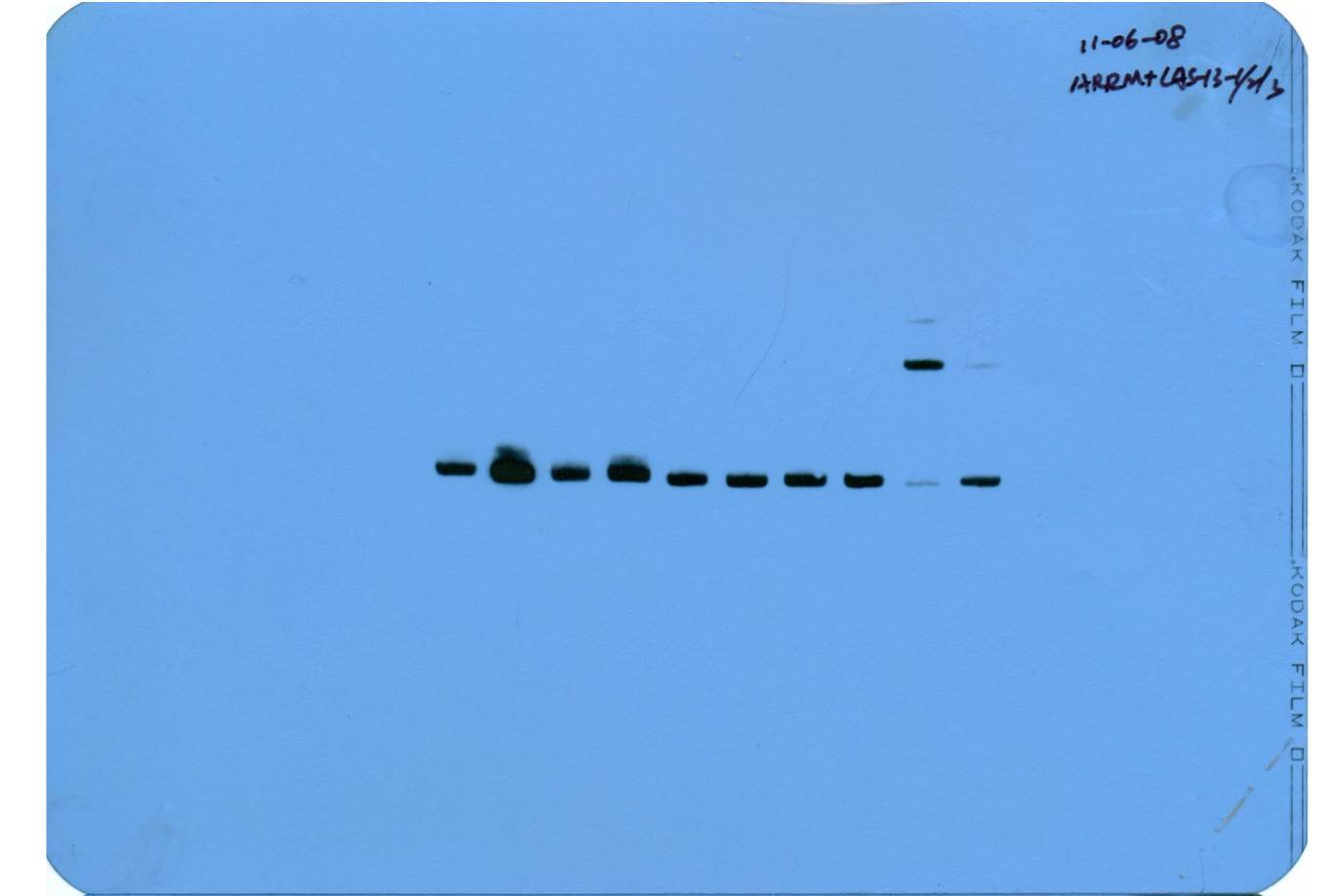

Supplement: Supplementary file 6 [file msb0010-0755-sd6.zip › Sources Data for Figure 4E/ARR1_pLAS-13-1_through_3.jpg]

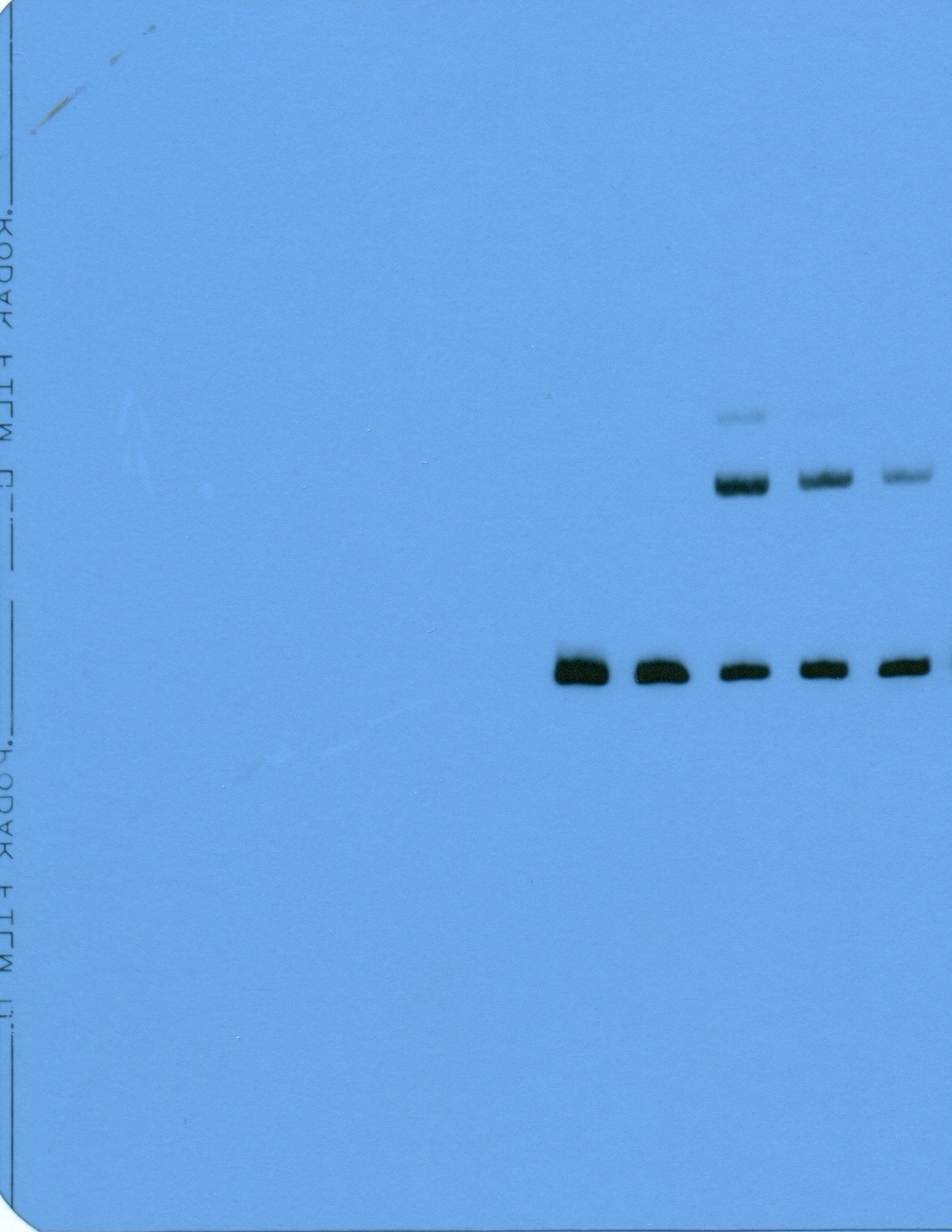

Supplement: Supplementary file 6 [file msb0010-0755-sd6.zip › Sources Data for Figure 4E/CUC2_pLAS-12-1.jpg]

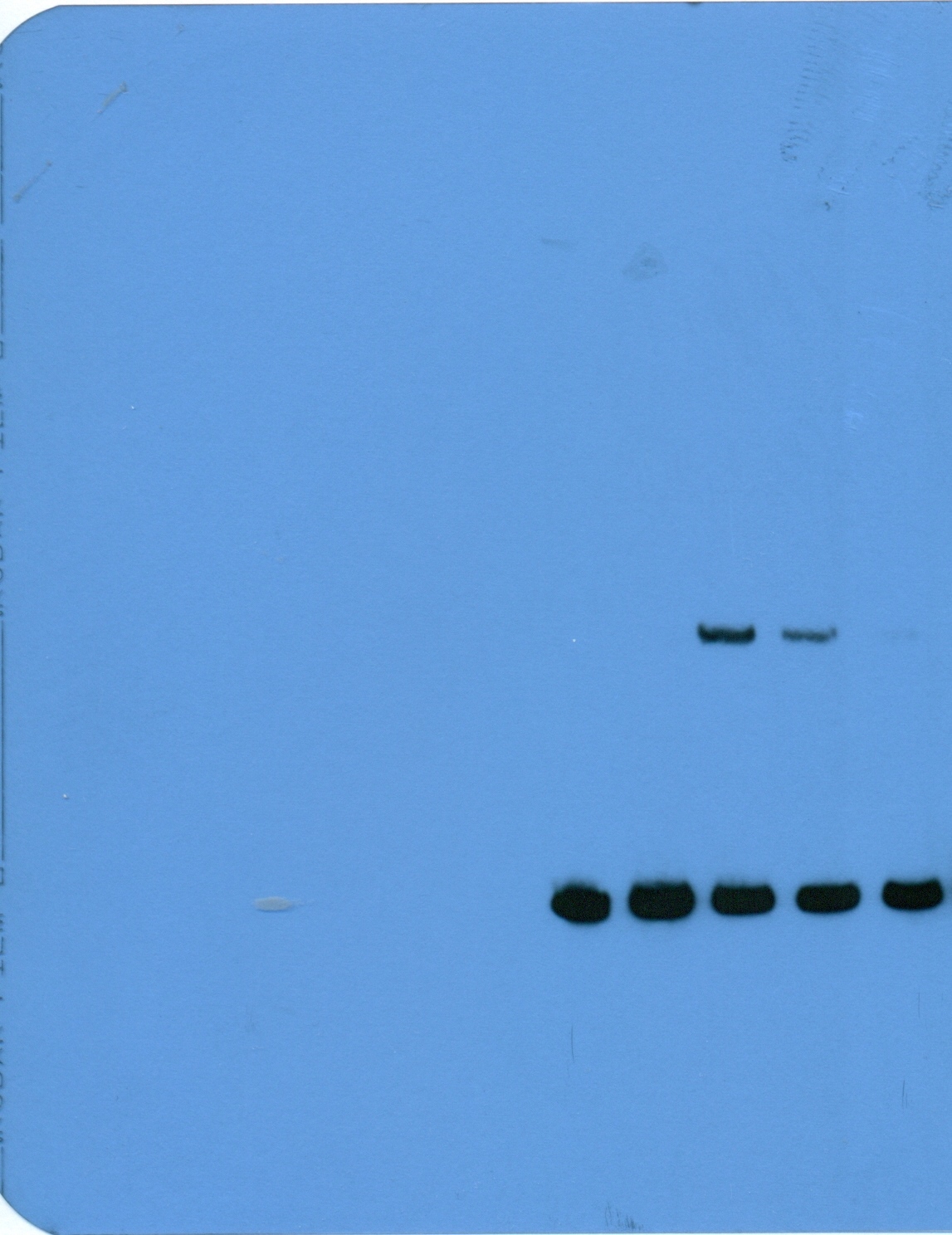

Supplement: Supplementary file 6 [file msb0010-0755-sd6.zip › Sources Data for Figure 4E/CUC2_pLAS-12-2.jpg]

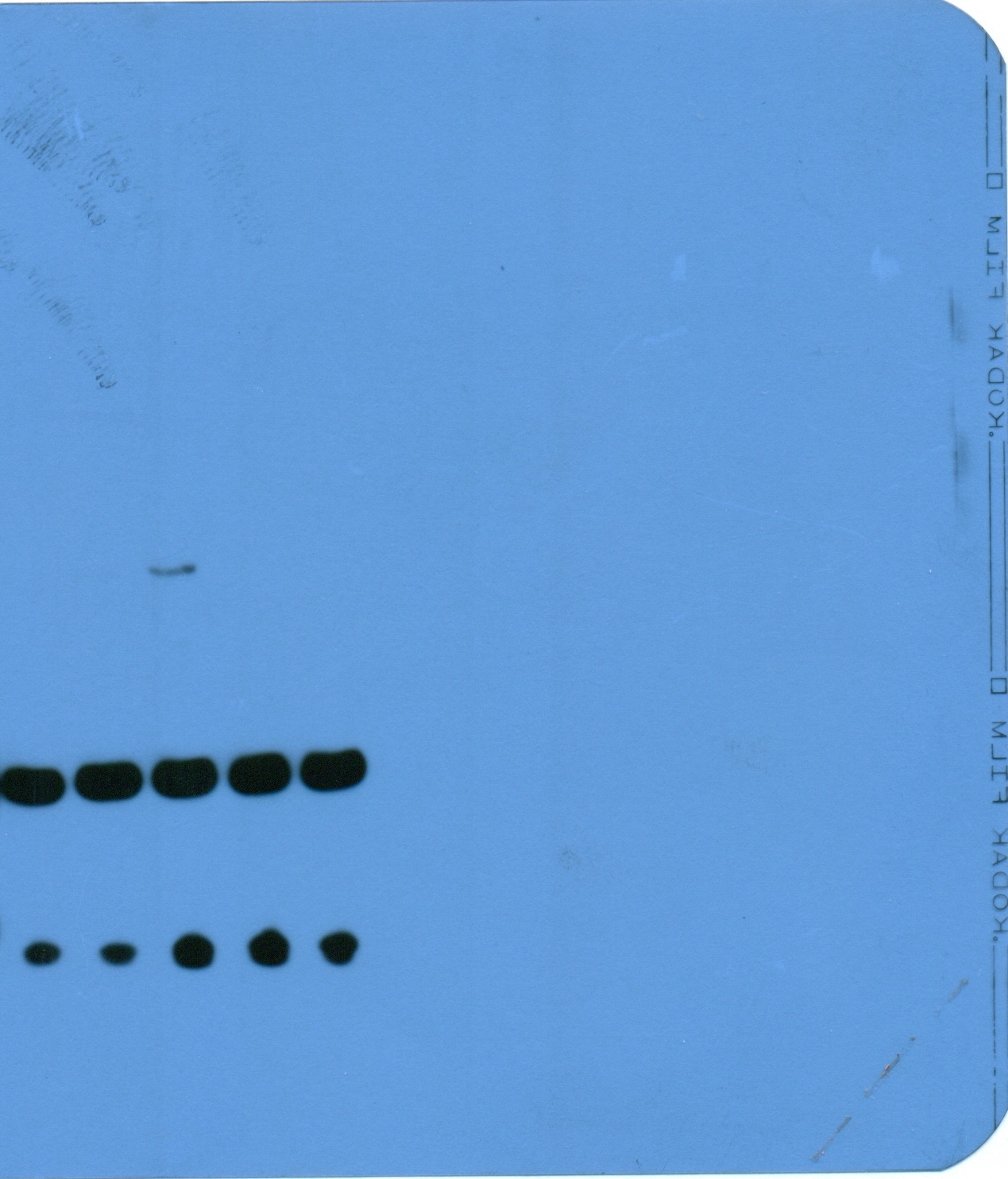

Supplement: Supplementary file 6 [file msb0010-0755-sd6.zip › Sources Data for Figure 4E/CUC2_pLAS-12-3.jpg]

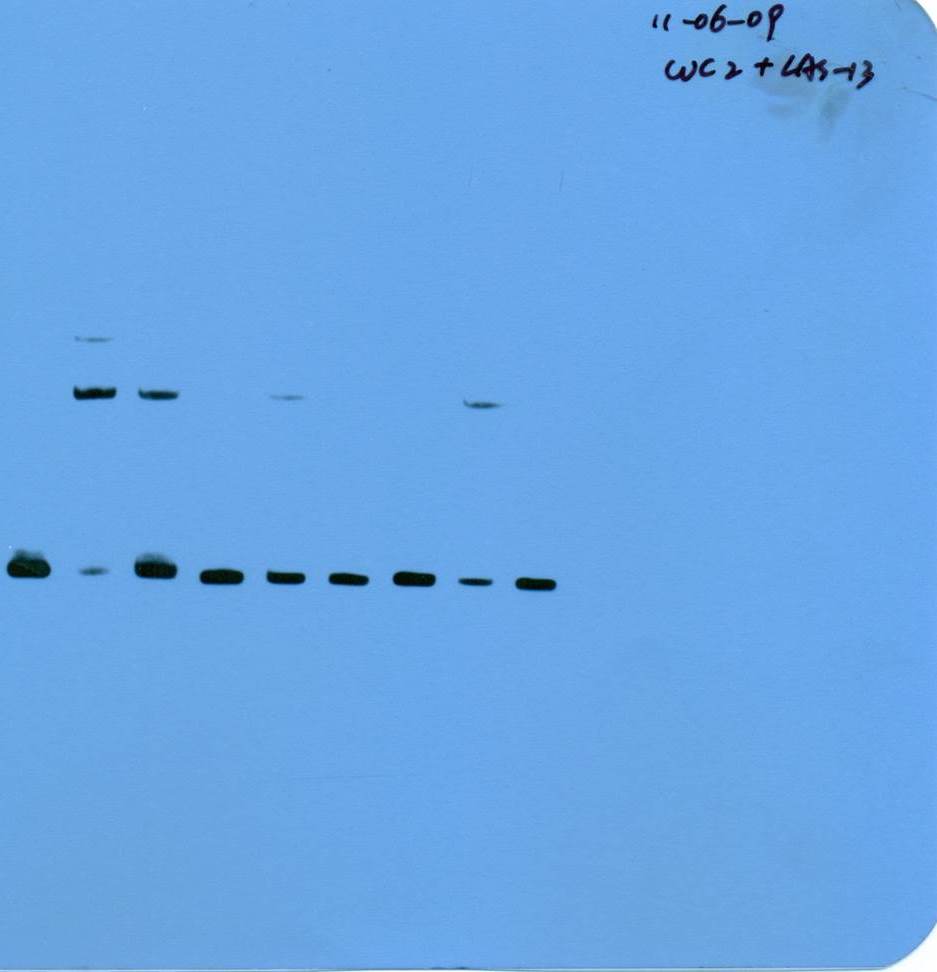

Supplement: Supplementary file 6 [file msb0010-0755-sd6.zip › Sources Data for Figure 4E/CUC2_pLAS-13-1_through_3.jpg]

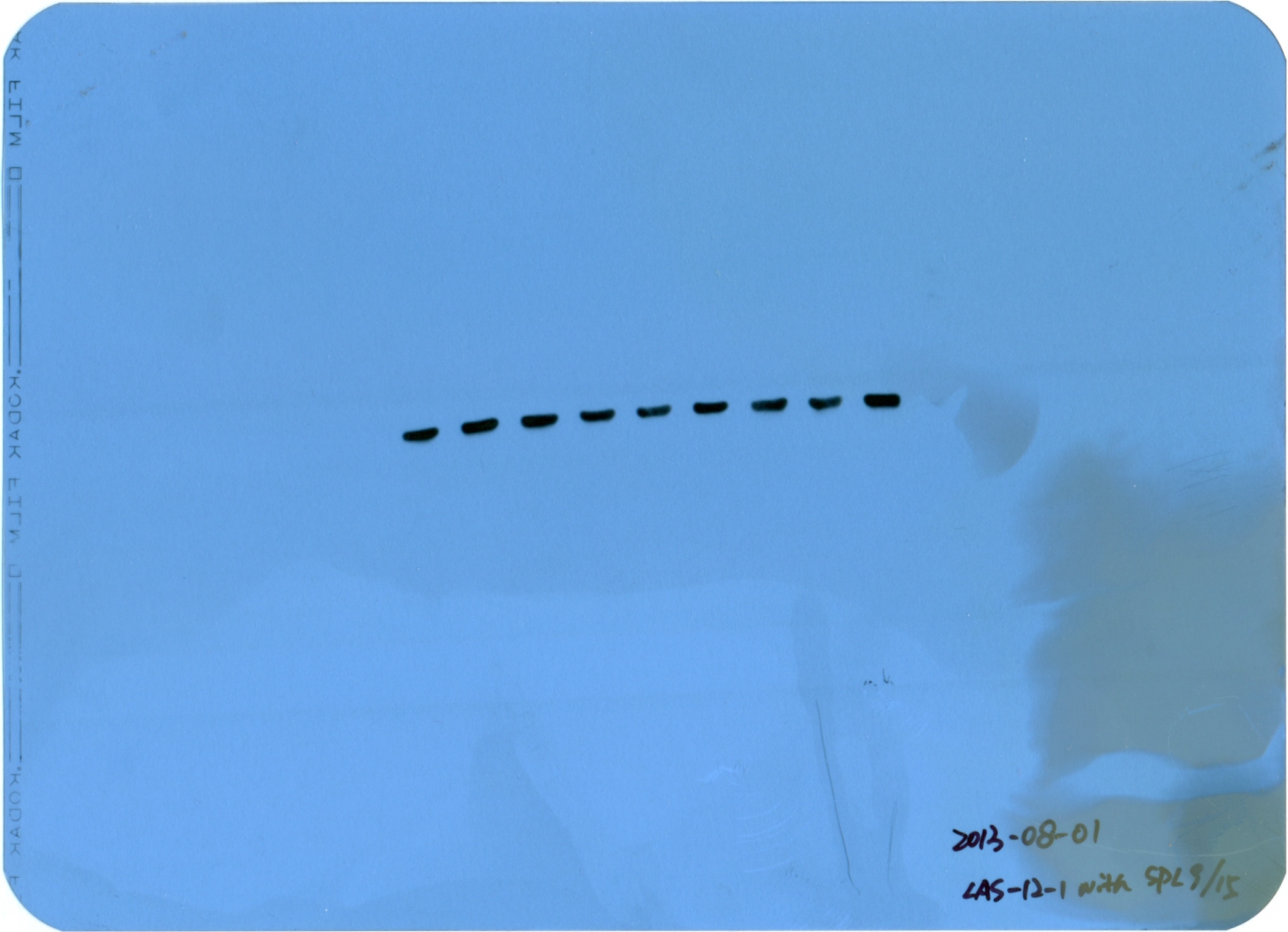

Supplement: Supplementary file 6 [file msb0010-0755-sd6.zip › Sources Data for Figure 4E/SPLs_pLAS-12-1.jpg]

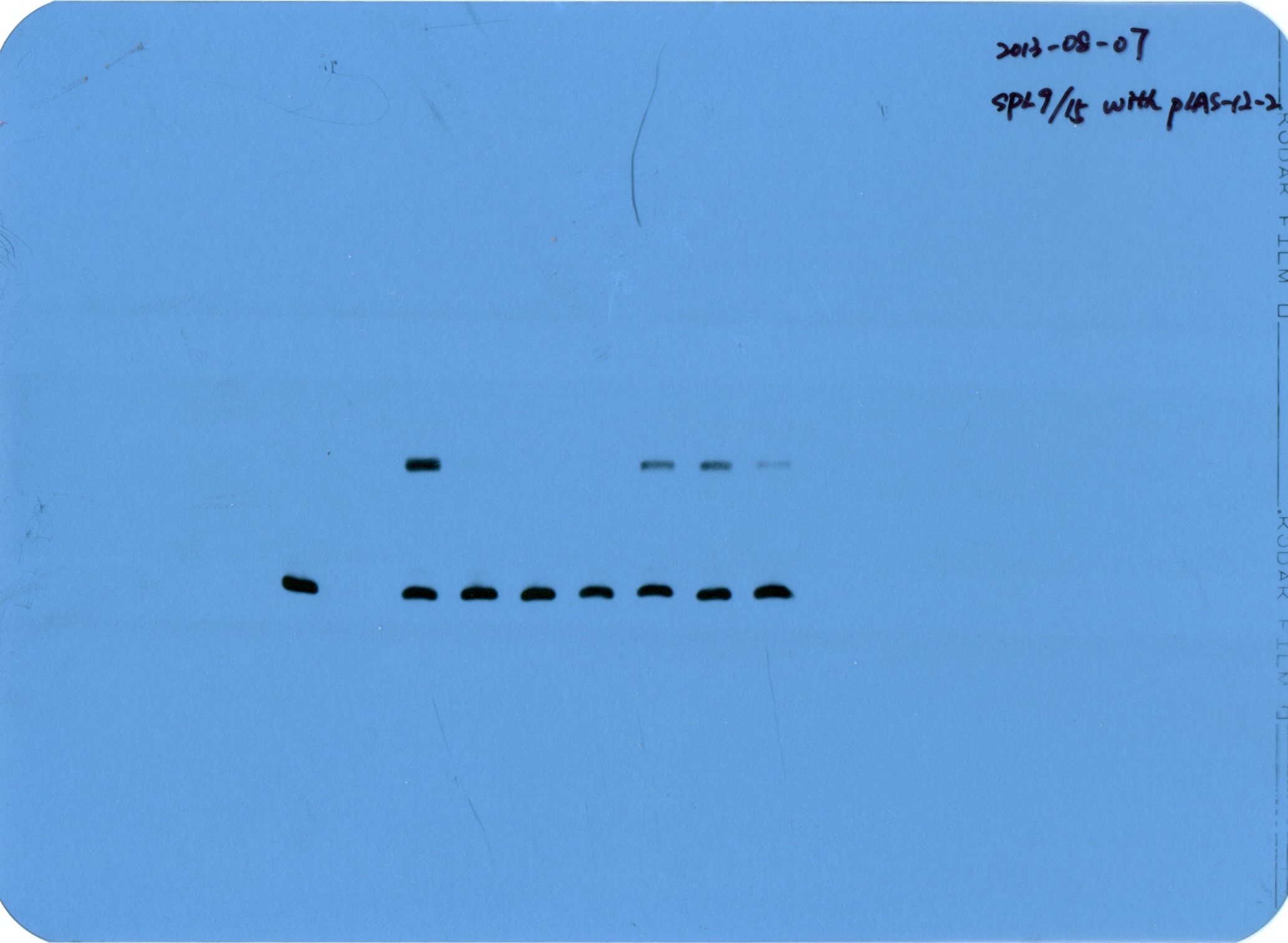

Supplement: Supplementary file 6 [file msb0010-0755-sd6.zip › Sources Data for Figure 4E/SPLs_pLAS-12-2.jpg]

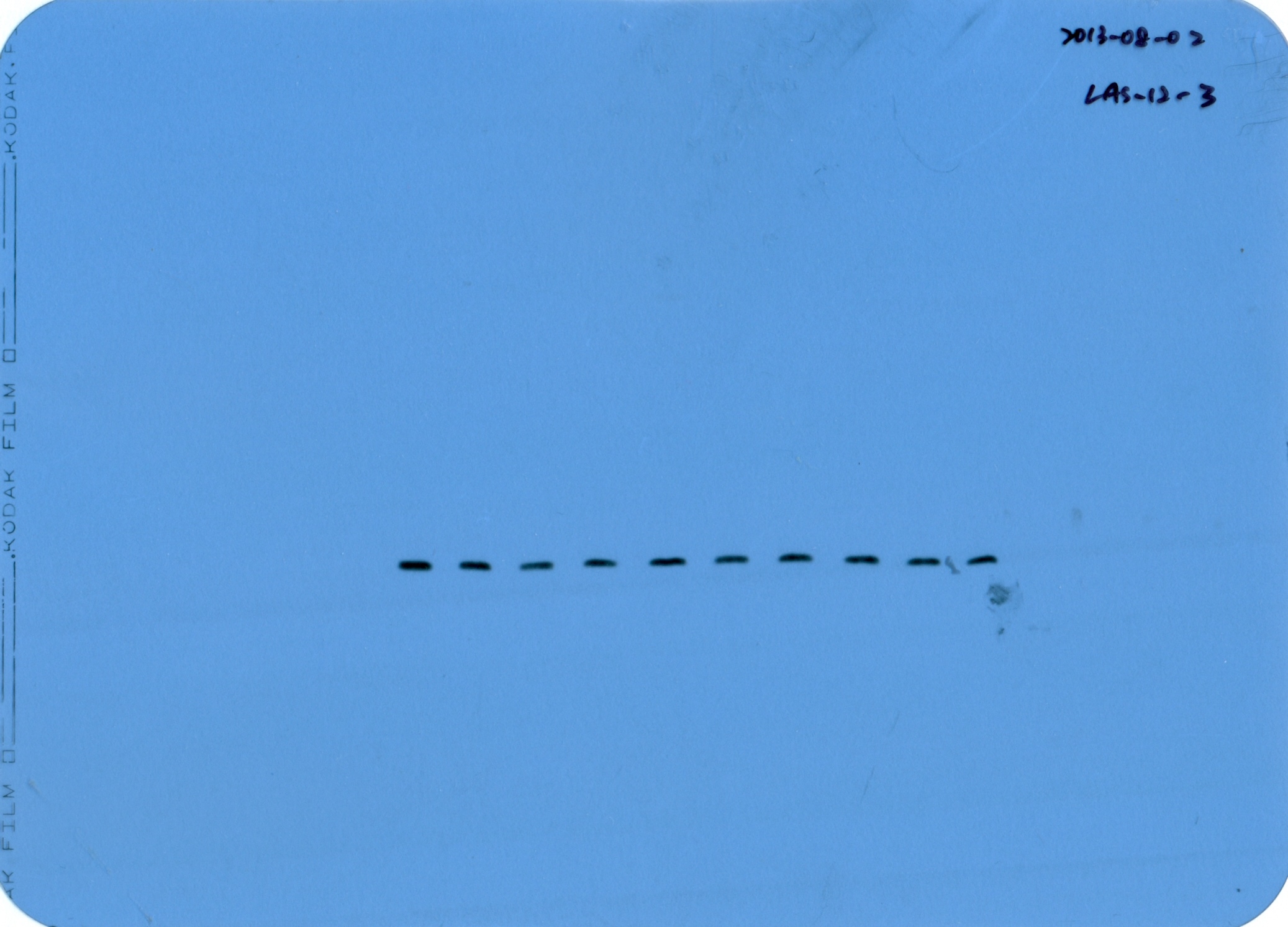

Supplement: Supplementary file 6 [file msb0010-0755-sd6.zip › Sources Data for Figure 4E/SPLs_pLAS-12-3.jpg]
