# Supplementary material for: An organ boundary-enriched gene regulatory network uncovers regulatory hierarchies underlying axillary meristem initiation
Source: Mol Syst Biol. 2014 Oct 30;10(10):1–2. doi: 10.15252/msb.20145470 (PMC4299377; doi:10.15252/msb.20145470)
Supplement: Supplementary file 3 [file msb0010-0755-sd3.pdf]

An organ boundary-enriched gene regulatory network uncovers regulatory hierarchies underlying axillary meristem initiation

Caihuan Tian, Xiaoni Zhang, Jun He, Haopeng Yu, Ying Wang, Bihai Shi, Yingying Han, Guoxun Wang, Xiaoming Feng, Cui Zhang, Jin Wang, Jiyan Qi, Rong Yu, Yuling Jiao

Corresponding author: Yuling Jiao, Institute of Genetics and Developmental Biology, Chinese Academy of Sciences

Review timeline:

Submission date:	17 June 2014
Editorial Decision:	18 July 2014
Revision received:	14 August 2014
Accepted:	24 September 2014

Editor: Thomas Lemberger

Transaction Report:

(Note: With the exception of the correction of typographical or spelling errors that could be a source of ambiguity, letters and reports are not edited. The original formatting of letters and referee reports may not be reflected in this compilation.)

1st Editorial Decision 18 July 2014

Thank you again for submitting your work to Molecular Systems Biology. We have now heard back from the three referees who accepted to evaluate the study. As you will see, the referees find the topic of your study of potential interest and are supportive. They make however a series of comments and make suggestions for modifications, which we would ask you to carefully address in a revision of the present work.

The most important points that should be concinvingly addressed are the following:
- when possible, the present dataset should be compared to existing similar datasets (eg Yadav et al 2014)
- the quality of the EMSA data should be improved, given the variable success of the competion experiments shown in this study.

With regard to the availability of the data presented in this study we would kindly ask you to address the following points:

- we appreciate that you include the accession number of the Trap-Seq data. Please provide also the full dataset of the Yeast 1-Hybrid assay. This data can be included in the supplementary information section as 'dataset' file.
- we would encourage you to provide the Cytoscape (or similar) file for the network representation shown in Figure 3C. This can be supplied as a "Source Data for Figure C3" file which will be directly downloadable from the figure.
- We would also encourage you to supply the full images of the original scans of each EMSA as 'source data' files (hand labeling of the lanes is fine, there is no need to provide any special layout).

Reviewer #1:

In this manuscript, Tian et al. utilize a gene regulatory network to identify key transcription factors that regulate organ boundary and axillary meristem formation in Arabidopsis. The mechanism by which organ boundaries are established and axillary meristems (AM) are initiated has remained a mystery, because cells in these regions are limited in number and related mutants can be difficult to identify. To overcome these limitations, Tian et al. utilized systems biology approaches to identify key regulators and network connections underlying these developmental processes.

The authors first utilized TRAP-seq to generate translatome profiles for the rare boundary and axillary meristem (leaf primordia) populations. This approach allowed the identification of unique translatome signatures for each of the two populations. In depth translatome analysis identified enriched TF family promoter motifs and unique hormone response signatures. Using yeast-1-hybrid, the authors focused on TF regulation of four gene promoters known to regulate boundary and AM formation. PDI identified from Y1H were then validated by EMSA and ChIP. When available, mutant, inducible or overexpression lines for TFs in the GRN were tested for regulation in planta. Next, the authors reasoned that mutation or overexpression of upstream TFs would exhibit similar phenotypes to the genes they regulate. Indeed this proved to be the case for several TFs, which the authors illustrate with boundary domain phenotypes. Together, theses results identify key connections regulating the boundary domain and AM initiation.

The body of work presented here represents a comprehensive establishment of the GRN regulating boundary domain and AM initiation in Arabidopsis. While several studies have generated GRN in Arabidopsis, the challenge remains to identify the biological significance of this network. Tian et al. do this impressively presenting a solid story beginning with translatome profiling and ending with validated interactions that regulate a developmental process. This work should be of interest to both plant biology and more general systems biology audiences.

Major points
None

Minor points
- In the discussion, the authors mention that "Molecular phenotypes for 73.3% of tested PDI", a few paragraphs later they say "identified expression phenotypes at the molecular level of 65.6% of TFs tested". Please clarify what you mean by a molecular phenotype versus an expression phenotype.

Reviewer #2:

This manuscript describes a thorough systems analysis of the transcriptional regulatory network that controls organ boundary formation. Combining a cell type specific expression analysis with a high throughput genome wide Y1H assay they have studied the main properties of the network, identifying dominant signatures and novel regulators of boundary function and axillary meristem formation.

This study is clearly of interest for those working in plant developmental biology and meristem function, which is currently a very active research field. The work is based on a solid technical know-how of the group and should provide a useful source for further work. In addition, the analysis has identified a number of previously unknown interactions.

In spite of this overall positive impression, there are some caveats. First of all, the manuscript is largely confirmatory and although it provides a solid basis for further understanding, it fails to give any real novel insight in the regulation of development. No novel global regulatory principles at the meristem (negative feedback or feedforward loops,etc...) are identified for instance. In principle such a detailed comparison between boundary and organ specific expression might have provided some insights in the fundamental differences between both domains, but this does not seem to be the case as the manuscript only provides very general statements on this issue. Boundary cells seem to express eg 'meristem initiation, 'DNA Binding', 'Hormone Stimulus', 'Histone Modification' genes, which in principle is not very surprising. Note that the enriched functions for 'organ development' and 'cell cycle' are less evident, as in boundary regions cells divide very slowly and organs are rather formed next to to the boundary..

I also performed a number of checks on the excel lists provided as supplementary files. Although I realise that this type of work is never 100% free of false negatives or positives, I was somewhat surprised to see that major genes involved in organ initiation and strongly expressed in organ primordia such as ANT or MP were absent from the boundary depleted list. In principle STM should also be enriched in boundaries vs organs, but I could not find it in the Supp Table 2. The authors should make sure that their analysis is as exhaustive as they claim.

The authors should also make sure that they compare their results to those obtained by others. Recently, Yadav et al (Development 2014) also used the LAS promoter to identify boundary specific transcripts for example. In principle they should find overlapping results. Is this indeed the case ?

In conclusion, provided the authors further confirm te quality of their analysis, this study provides an exhaustive database on a fundamental aspect of plant developmental biology. As it does not (yet) lead to profound novel insights in the overall properties of the GRNs involved, it will be a matter of editorial policy if this type of useful analysis is acceptable for MSB.

Reviewer #3:

This m/s describes a gene regulatory network enriched in boundary cells that will give rise to axillary meristems. The authors analyse:

1. Expression in enrichted tissues.
2. Upstream regulators of key components in axillary meristems (selected based on previous knowledge).
3. Validation of binding of upstream regulators and of their transcriptional regulatory effect.
4. Validation of biological significance of new components of the inferred gene regulatory network.

In general, the experimental evidence for the inferred network is convincing. It is particularly reassuring that the authors reestablish the importance of several known factors in axillary meristem development and that they bolster very recent work on the role of hormone signal transduction in these regions. The authors also validate experimentally a number of gene functions which they newly implicate in the axillary meristem GRN. We think that this is one of the strenghts of this manuscript, as it demonstrates that their approach is useful for rapid extension of gene regulatory network function.

Being a collection of high-throughput analyses, there are some points where the information presented is not sufficient to fully support the conclusions. Prior to acceptance, these shortcomings need to be addressed:

1. In the EMSA analyses reported in Fig. 4, several band shifts are not competed with excess unlabeled probe, unlike stated by the authors. Also, some band shifts reveal no free probe whereas other flanking ones do, suggesting that the amount of input was different. Both these issues need to be clarifed.

2. The raw data for Figure 3 are not shown in the figure - Fig S3 is hard to interpret and in our view does not comprehensively report on the experiment. As this is a key figure in the paper, with information that will likely be used by a larger research community, a more elaborate representation of these data is warranted.

3. Fig. S4 needs a more elaborate explanation.

Minor point: the GO analyses described in Fig 2 seem over-interpreted. In our view, their major use is to bolster the separation of tissue types.

1st Revision - authors' response 14 August 2014

Point-by-point Response to the Reviewers' Comments

We would like to thank the editor and the reviewers for the careful and constructive reviews. Based the comments from the editor and the reviewers, we have made changes of the manuscript, which are detailed below.

Editor

1) When possible, the present dataset should be compared to existing similar datasets (eg Yadav et al 2014)

Reply: Thank you for pointing out this related new publication to us. We compared our dataset with the Yadav et al. at 2014 paper, and identified significant overlapping between lists of genes enriched in corresponding cell types. Although different tissues (seedlings vs. floral meristems, FMs) and different comparison methods were used, we found that 38 out of the 144 *LAS*-enriched FM genes (when compared to *CLV3* and *KAN1* domains, identified by the Yadav et al. paper) were also found enriched in the seedling *LAS* domain. This enrichment is highly significant with a $P < 7.98E-36$ using the hypergeometric test. When we compared *LAS*-enriched FM genes vs *CLV3*, *FIL* and *KAN1* domains identified in the Yadav et al. paper, we found 14 out of 58 were found in our *LAS*-enriched seedling dataset, which is again highly significant ($P < 3.38E-14$). We have included this comparison in the second paragraph in Page 9, and have added a new Expanded View Table E5 for detailed comparison results. As shown in Table E5, *LAS*-enriched genes identified by both Yadav et al. and us include well-identified boundary-specific genes *CUC3*, *LAS* and *LSH4*. In addition, we also recovered other reported boundary-specific genes, including *CUC1*, *CUC2*, *RAX1*, *BOP1*, *KNAT1*, *KNAT6*, *LBD13*, *LBD19*, *LBD16* and *LBD40* in our seedling *LAS* domain-enriched gene list.

2) The quality of the EMSA data should be improved, given the variable success of the competion experiments shown in this study.

Reply: We have repeated some of the EMSA experiments with further optimized protein/DNA probe ratio. We have replaced Fig 4B and E with new film scan images. As shown in the new figure panels, we obtained good competition results when adding unlabeled DNA probes for all tested PDIs, except between MBP-CUC2 and fragment *pLAS-13*. Nevertheless, this weak EMSA interaction was further verified by dissecting *pLAS-13* into three overlapping fragments, shown in Fig 4E. Original scans are also provided in Source Data for Figure 4B and E.

3) We appreciate that you include the accession number of the Trap-Seq data. Please provide also the full dataset of the Yeast 1-Hybrid assay. This data can be included in the supplementary information section as 'dataset' file.

Reply: We have already provided the full dataset of the Yeast 1-Hybrid assay in now Table E10, which includes each PDI, its confirmation result if available, regulatory potential if available, and corresponding TF expression. In addition, a detailed list of all TF preys was provided in now Table E8, and detailed information of all promoter fragments were shown in now Table E9. Please refer to our original uploaded files if the converted PDF file omits any part.

4) We would encourage you to provide the Cytoscape (or similar) file for the network representation shown in Figure 3C. This can be supplied as a "Source Data for Figure C3" file which will be directly downloadable from the figure.

Reply: We have now included the cytoscape file for Fig 3C as Source Data for Figure 3C.

5) We would also encourage you to supply the full images of the original scans of each EMSA as 'source data' files (hand labeling of the lanes is fine, there is no need to provide any special layout). To facilitate the upload these files as a single zip archive, you can label

each image file with a filename that indicates which assay (eg ARR1-pLAS13) it corresponds to and group the images in folders labeled according to the individual panels (panel B, panel E) to which the images belong. These folders can then simply be included in a single top level folder which can be compressed as zip archive and uploaded as 'source data file for figure 3'.

Reply: We have now included original scans of all EMSA as a single zip archive file, which is labelled as Source Data for Figure 4B and E.

Reviewer 1

1) In the discussion, the authors mention that "Molecular phenotypes for 73.3% of tested PDI", a few paragraphs later they say "identified expression phenotypes at the molecular level of 65.6% of TFs tested". Please clarify what you mean by a molecular phenotype versus an expression phenotype.

Reply: Thank you for pointing this out. We indeed had the second percentage incorrect. It should be 73.3%, i.e. 22 out of 30 genes, for both places. We apologize for this mistake, and have revised the text in the last paragraph in Page 27.

Reviewer 2

1) First of all, the manuscript is largely confirmatory and although it provides a solid basis for further understanding, it fails to give any real novel insight in the regulation of development. No novel global regulatory principles at the meristem (negative feedback or feedforward loops,etc...) are identified for instance. In principle such a detailed comparison between boundary and organ specific expression might have provided some insights in the fundamental differences between both domains, but this does not seem to be the case as the manuscript only provides very general statements on this issue.

Reply: We appreciate that this reviewer considered our manuscript “provides a solid basis for further understanding”. In addition, we believe that our work also identifies new regulations in addition to confirmations. In this work, we did rediscover a number of known regulators for boundary and axillary meristem development. Together with the rediscovery, we identified regulatory principles of related upstream TFs and downstream genes (Fig 8), which are novel. We also tested regulatory potentials of a substantial number of PDIs identified in this work (Fig 6B). Regarding phenotypic characterization, we also characterized 36 mutant/overexpressing alleles, and identified several genes with novel boundary phenotypes, such as *DRN*, several *SPL*s, and *HDG12*.

We agree that more regulatory principles are likely to exist in the regulation of boundary and axillary meristem development. The current work mostly focuses on high-throughput genome-wide identification of new regulations and new regulators, and more detailed regulatory principles require further modeling and experimental test of selected regulations. In fact, more focused studies on some of the PDIs reported here by us and other colleagues in this field are ongoing. We also agree that boundary domain cells are fundamentally different from leaf cells, although they are next to each other. For instance, we have seen very different phytohormone responses between these two groups of cells (Fig 2D). Among them, domain-specific auxin and cytokinin signaling confirms more focus mechanistic studies by us and others (Han et al., 2014, *PNAS* 111:6840-6845; Wang et al., 2014, *Plant Cell* 26:2055-2067; Wang et al., 2014, *Plant Cell* 26:2068-2079). This current work further extends the recent reported cytokinin regulation by identifying direct interaction between the *LAS* promoter and ARR1, a cytokinin signaling component. Because of the focus of this current manuscript, we are unable to test all potential regulations in details, and would like to allow further studies by the community to test, and hopefully confirm, potential regulatory principles identified by our genome-wide approaches, such as cell cycle, histone methylation, cell wall constituents, etc.

2) Boundary cells seem to express eg 'meristem initiation, 'DNA Binding', 'Hormone Stimulus', 'Histone Modification' genes, which in principle is not very surprising. Note that the enriched functions for 'organ development' and 'cell cycle' are less evident, as in boundary regions cells divide very slowly and organs are rather formed next to to the boundary..

Reply: We were glad to see that some expected GO terms were recovered from our cell type-specific translatome profiling and from Y1H identification of PDIs, because that confirmed the reliability of our genome-wide assays. GO term ‘cell cycle’ includes both positive and negative regulators of cell cycle, and for boundary domain cells, more negative regulators are enriched. We have further explained this situation in the first paragraph in Page 10.

3) I also performed a number of checks on the excel lists provided as supplementary files. Although I realise that this type of work is never 100% free of false negatives or positives, I was somewhat surprised to see that major genes involved in organ initiation and strongly expressed in organ primordia such as ANT or MP were absent from the boundary depleted list. In principle STM should also be enriched in boundaries vs organs, but I could not find it in the Supp Table 2. The authors should make sure that their analysis is as exhaustive as they claim.

Reply: Thank you for your comments. We were also surprised to notice that both *ANT* and *MP* were excluded from the boundary depleted gene list. To explicitly see their expression patterns, we used confocal imaging of a *pANT::LhG4 pOp::GFP-ER* line (Schoof et al., 2000, *Cell* 100:635-644) and a *pMP::MP-GFP* line (Schlereth et al., 2010, *Nature* 464:913-916). As shown in the longitudinal cross section through the SAM region, strong GFP signal was found in the boundary region, suggesting strong *ANT* expression. In addition, *ANT* promoter activity was found only in young leaf primordia but not mature leaves older than P_{12}, which is contrast to *AS1* expression in mature leaves and cotyledons (Fig E1). On the other hand, *ANT* expression persists in older leaf axils. Therefore, *ANT* is not depleted from the boundary domain. *MP* also

has stage-specific expression with enrichment in young leaf primordia but not mature leaves. In addition, *MP* expression also extends into the boundary region, although to a weaker extend than *ANT*. Therefore, *MP* is not excluded from boundary either, which is a bit contradicting to what people would assume. Because boundary cells can respond to auxin (Wang et al., 2014, *Plant Cell* 26:2055-2067; Wang et al., 2014, *Plant Cell* 26:2068-2079), it is expected that *MP* or other ARF(s) with transcriptional activating function (i.e. ARF6, 7, 8, or 19) has boundary expression. In our dataset, we found *STM* was slightly below our boundary-enrichment cutoff (logFC > 1 and P < 0.001) with logFC = 0.88 and P = 0.022. In addition to boundary-enriched expression, *STM* also has vascular expression (Figs 3 and 4 in Grbić and Bleecker, 2000, *Plant J* 21:215-223). The high abundance of vasculature tissues within the *AS1* expression domain may explain the observed weak enrichment of *STM* in boundary cells.

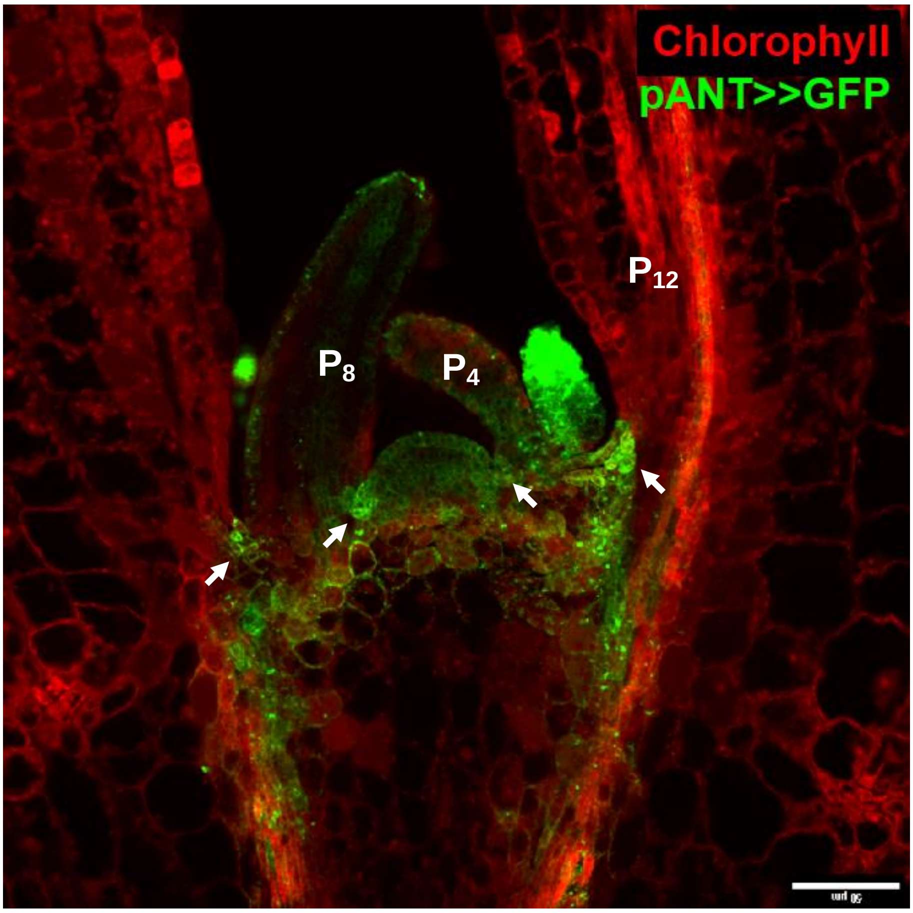

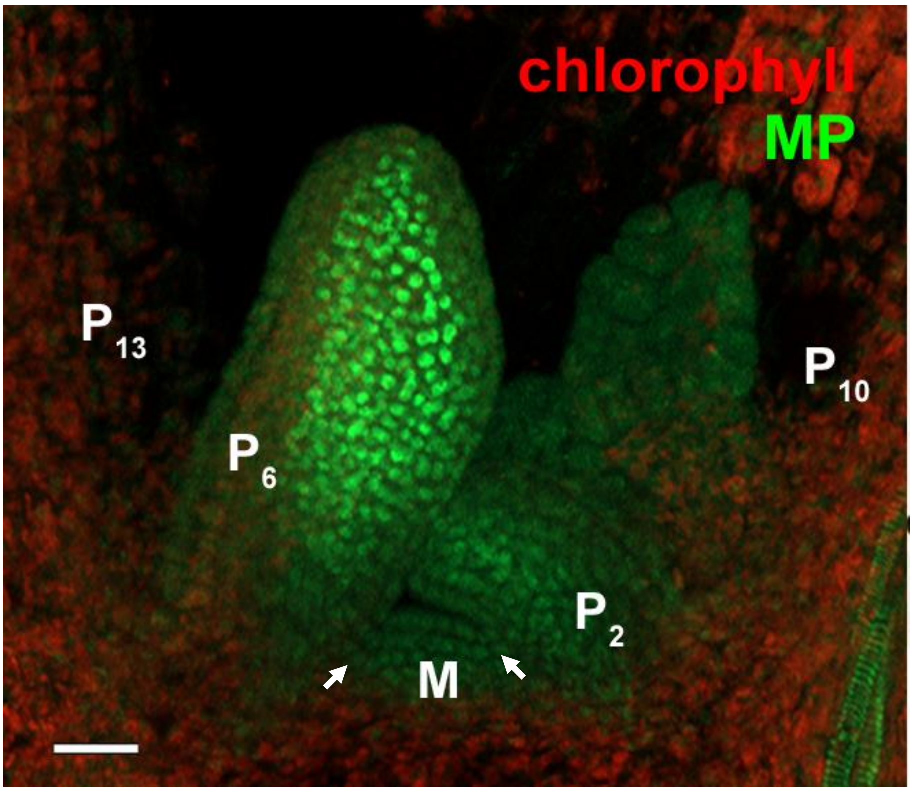

4) The authors should also make sure that they compare their results to those obtained by others. Recently, Yadav et al (Development 2014) also used the LAS promoter to identify boundary specific transcripts for example. In principle they should find overlapping results. Is this indeed the case ?

Reply: Thank you for pointing out this related new publication to us. We have compared our results with the Yadav et al. dataset. Please refer to above Reply to Editor' Comment 1 for details.

Reviewer 3

1) In the EMSA analyses reported in Fig. 4, several band shifts are not competed with excess unlabeled probe, unlike stated by the authors. Also, some band shifts reveal no free probe whereas other flanking ones do, suggesting that the amount of input was different. Both these issues need to be clarifed.

Reply: We have further optimized our EMSA assay, and replaced several images in Fig 4. Please refer to above Reply to Editor' Comment 2 and Fig 4B and E for details.

2) The raw data for Figure 3 are not shown in the figure Fig S3 is hard to interpret and in our view does not comprehensively report on the experiment. As this is a key figure in the paper, with information that will likely be used by a larger research community, a more elaborate representation of these data is warranted.

Reply: All raw data for Fig 3 were provided in now Table E8-E10. Please refer to above Reply to Editor' Comment 3 and these Tables for details. Figure E3 shows only one example of our Y1H assay to highlight how we mixed 4 TFs in each pool as one well, and carried out 96-well plate format Y1H assay. For those positive wells, we separated the corresponding four TFs and performed another round of Y1H assay to further identify which TF binds to the promoter tested. Detailed description of our pooling strategy can also be found in Pages 32 and 33.

3) Fig. S4 needs a more elaborate explanation.

Reply: Thank you for pointing this out. We have supplemented the figure legend to better explain this figure.

4) Minor point: the GO analyses described in Fig 2 seem over-interpreted. In our view, their major use is to bolster the separation of tissue types.

Reply: Thank you for your comment. We have revised our description about the GO analysis in the first paragraph in Page 10.
